# Mind the Gut: Cognitive Decline, Microbiota, and Nutrition-Related Modulators in Older Adults with Chronic Kidney Disease

**DOI:** 10.3390/nu18121978

**Published:** 2026-06-18

**Authors:** Lisa Bevilacqua, Federica Lenci, Leonardo Biscetti, Belinda Giorgetti, Robertina Giacconi, Marta Balietti

**Affiliations:** 1 Biogerontology Center, IRCCS INRCA, 60121 Ancona, Italy; b.giorgetti@inrca.it (B.G.); r.giacconi@inrca.it (R.G.); 2 Unit of Nephrology and Dialysis, IRCCS INRCA, 60126 Ancona, Italy; f.lenci@inrca.it; 3 Neurology Unit, IRCCS INRCA, 60128 Ancona, Italy; l.biscetti@inrca.it

**Keywords:** chronic kidney disease, aging, cognitive impairment, hemodialysis, gut microbiota, nutritional interventions

## Abstract

Chronic kidney disease (CKD) is a progressive condition characterized by persistent kidney abnormalities with systemic consequences. Beyond its metabolic and cardiovascular complications, CKD has been associated with structural and functional brain alterations that are particularly evident in advanced stages and in patients undergoing hemodialysis (HD). Deficits across multiple cognitive domains are frequently observed and may compromise treatment adherence, clinical management, and quality of life, yet remain largely underrecognized in clinical practice. Older adults are particularly vulnerable. Age-related brain changes and comorbidities may increase susceptibility to CKD-related cerebral alterations, while reduced cognitive reserve may amplify clinical impact. The gut–kidney–brain axis has emerged as a relevant biological pathway, with CKD-related dysbiosis potentially influencing inflammation, metabolic homeostasis, and the generation of uremic metabolites linked to neurological dysfunction. This review examines the mechanisms contributing to brain vulnerability in older adults with CKD, with specific attention to patients undergoing HD, and discusses challenges in the recognition and assessment of cognitive impairment in this population. It further explores microbiota-targeted nutritional strategies as potentially modifiable approaches to modulate gut-derived metabolic and inflammatory processes relevant to brain health, although current evidence for direct effects on cognitive outcomes remains limited.

## 1. Introduction

**Figure 1:**
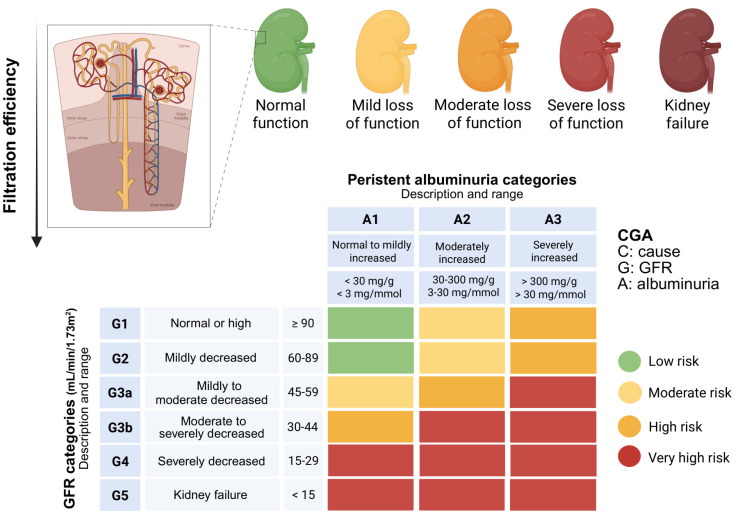
CGA classification of chronic kidney disease. The CGA framework integrates the underlying cause (C), glomerular filtration rate (GFR; G), and albuminuria (A). GFR reflects the level of kidney function, while albuminuria represents a marker of kidney damage and increased glomerular permeability. The figure is based on KDIGO (Kidney Disease: Improving Global Outcomes) guidelines [2] and was created using BioRender.

Chronic kidney disease (CKD) is defined by the presence of structural or functional kidney abnormalities persisting for at least three months, with implications for health [1,2]. These alterations may lead to a range of clinical consequences, including electrolyte disturbances, cardiovascular complications, and an increased risk of progression to kidney failure requiring renal replacement therapy, such as dialysis or transplantation. CKD is currently classified according to the CGA framework (Figure 1).

CKD affects approximately 788 million people worldwide (~14% of the global population) and is among the fastest-growing causes of death, projected to rank as the fifth leading cause of mortality by 2050 [3,4]. The prevalence of CKD is particularly pronounced in older adults (≥65 years), with estimates ranging from 34% [5] to 44% [6], depending on study design, diagnostic criteria, and cohort characteristics. The higher prevalence in older adults is further compounded by frequent multimorbidity, which complicates CKD diagnosis and management. A well-recognized example is sarcopenia, an age-related loss of muscle mass that can lower circulating creatinine, a muscle-derived metabolite commonly used to estimate glomerular filtration rate (GFR) [7], thereby potentially overestimating kidney function. This overestimation may lead to inappropriate dosing of renally cleared drugs and increased risk of toxicity [8], as well as misclassification of disease severity [2].

CKD is characterized by several direct complications, including highly prevalent conditions such as anemia [9] and secondary hyperparathyroidism [10]. CKD has also been associated with an increased risk of both ischemic and hemorrhagic events [11] and with a higher burden of cognitive decline [12]. Cognitive impairment in the absence of overt dementia is common and may affect up to 50% of patients in advanced stages [13,14]. However, it remains substantially underrecognized in routine clinical practice. In older adults, this may have a significant impact, especially in those with reduced cognitive reserve [15].

Emerging evidence suggests that CKD-associated disturbances in the gut microbiota may contribute to disrupted kidney–brain communication [16], with potential effects on brain homeostasis and function. Age-related changes in the gut microbiota [17,18] may further influence these processes, underscoring older adults with CKD as a particularly vulnerable and clinically relevant population.

This review examines the mechanisms contributing to cognitive decline in older adults with CKD, with specific attention to patients undergoing hemodialysis (HD), and discusses current challenges in recognizing cognitive impairment, with emphasis on tailored strategies that may support its early detection in clinical practice. Nutritional interventions aimed at modulating the gut microbiota are explored as potentially modifiable tools for targeting microbiota-related metabolic and inflammatory pathways involved in kidney–brain interactions and cognitive vulnerability.

This is a narrative review, and the following principles were used to guide literature selection. PubMed was searched using combinations of terms including “chronic kidney disease” or “CKD”, “dialysis” or “hemodialysis”, “ageing” or “ageing”, “older adults”, “cognitive impairment”, and “dysbiosis”. No formal date restriction was applied, although priority was given to articles published within the last 10 years when available. Only articles written in English were considered. Clinical, preclinical, and review articles were included according to their relevance to each topic, and the nature of the evidence was specified in the text where appropriate.

## 2. Neurological Changes in CKD

**Figure 2:**
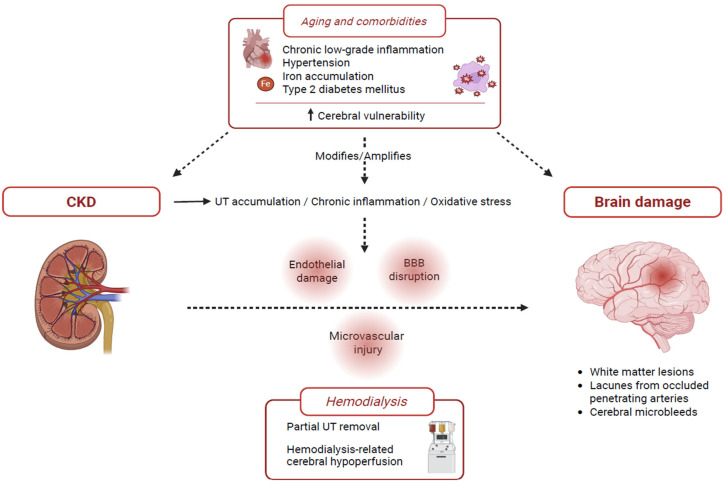
Cerebral vulnerability in chronic kidney disease (CKD) and aging. CKD contributes to brain vulnerability through multiple interconnected pathways, including vascular injury, uremic toxin (UT) accumulation, and dialysis-related factors. Aging acts as a cross-cutting modifier, amplifying these mechanisms through increased microvascular burden, inflammation, and metabolic alterations. Solid arrows represent consequences or main proposed mechanistic pathways, whereas dashed arrows indicate indirect, modifying, or not directly causal associations. BBB, blood–brain barrier. Created using BioRender.

Neurological abnormalities in CKD have long been recognized following seminal work in the field [19,20] and are associated with multiple pathophysiological mechanisms, selected examples of which are summarized here (Figure 2).

Both the kidney and the brain are low-resistance organs exposed to high blood flow and dependent on intrinsic autoregulation, making them particularly susceptible to microvascular dysfunction [21]. In patients with CKD, vascular injury represents a major pathway of cerebral damage. The accumulation of uremic toxins (UTs) and the establishment of a chronic proinflammatory milieu promote endothelial dysfunction, blood–brain barrier (BBB) impairment, and microvascular damage [22,23,24]. These alterations may contribute to the development of white matter lesions, lacunes from occluded penetrating arteries, and cerebral microbleeds [25,26,27,28]. Enlarged perivascular spaces are also observed in patients with CKD, likely reflecting impaired glymphatic and perivascular fluid transport and reduced interstitial waste clearance [29]. In older adults, these features may be particularly pronounced due to the high prevalence of cardiovascular comorbidities, such as hypertension and type 2 diabetes mellitus, which contribute to cumulative damage [30,31].

Beyond vascular mechanisms, other processes contribute to neuronal vulnerability in the context of kidney failure. For instance, in CKD patients, particularly those undergoing HD, iron accumulates excessively in specific brain regions, leading to increased oxidative stress [32]. In older adults, this CKD-related accumulation adds to the abnormal iron deposition already typical of aging [33]. Other factors that may amplify the combined effects of aging and CKD include sustained neuroimmune activation and the redox imbalance characteristic of the aged brain [34,35]. Indeed, UTs exert direct neurotoxic effects by activating inflammatory signaling pathways and promoting oxidative stress [36,37,38,39].

Dialysis-related factors may also contribute to neurological injury in CKD. Conventional HD efficiently removes small UTs (<500 Da) but is less effective at clearing middle molecules and protein-bound toxins [40]. High-flux HD and hemodiafiltration improve middle molecule clearance and have been associated with better outcomes [41,42], although their availability remains limited. Expanded HD, which can enhance clearance of middle to large UTs, may further improve toxin removal; however, its long-term safety and efficacy remain to be established [43]. Nonetheless, dialysis itself may also contribute to neurological vulnerability. Rapid urea removal can cause dialysis disequilibrium syndrome, leading to osmotic imbalance and cerebral edema [44]. Notably, in older patients, HD sessions are associated with reduced cerebral blood flow, potentially leading to recurrent hypoperfusion and ischemic brain injury [45]. Recent CKD-specific evidence further supports this mechanism: in a prospective cohort of older patients undergoing HD, greater intradialytic cerebral blood flow reductions were associated with worsening global cognition, executive function, and memory over 12 months [46]. In this regard, some strategies, including the control of ultrafiltration rate and the adjustment of the sodium concentration of the dialysis fluid, can be very useful in order to reduce the risk of HD harmful effects on brain health [47].

## 3. Epidemiological Evidence Linking Cognitive Decline with CKD and HD

CKD has been associated with an increased risk of cognitive decline, with studies reporting up to a 65% higher likelihood of cognitive deficits [48], although clinical manifestations are heterogeneous across patients. Meta-analyses indicate that cognitive impairment is most prominent in patients with end-stage kidney disease (ESKD) receiving HD and involves orientation, memory, attention, information processing, language, visuospatial abilities, executive functions, and subjective cognitive complaints [49,50,51], consistent with a multidomain pattern of cognitive dysfunction in this population. The risk of cognitive impairment is higher in older adults, women, and patients with a history of stroke [52]. Notably, cognitive impairment increases progressively with age: in a Japanese cohort of CKD patients on HD, the prevalence of impairment based on Mini-Mental State Examination (MMSE) scores increased from 16% in those aged 60–69 years to 29% in those aged 70–79 years and reached 32% in individuals aged 80 years and older [53].

Although cognitive changes can compromise self-management and treatment adherence [54,55], leading to higher rates of hospitalization and mortality [56] and reduced quality of life [57], this condition remains largely underrecognized in clinical practice and is documented in fewer than 15% of affected cases [58].

To date, the most comprehensive assessment of cognitive function in CKD patients undergoing HD was conducted in the COGNITIVE-HD study [59], which applied an extensive neuropsychological battery, including Rey Auditory Verbal Learning Test (immediate and delayed recall), Symbol Digit Modalities Test, Digit Span Forward, Digit Span Backward, F-A-S Phonemic Fluency, RBANS (Repeatable Battery for the Assessment of Neuropsychological Status) Semantic Fluency, RBANS Picture Naming, RBANS Line Orientation, and RBANS Figure Copy. Drew and colleagues [60] later proposed an alternative battery, including the MMSE and a modified MMSE, the Montreal Cognitive Assessment (MoCA), the Trail Making Test Part B, the Mini-Cog Test, and the Digit Symbol-Coding Test, which assesses fewer domains but may be more feasible in routine clinical practice. Two recent studies have further explored this area by using the Memory Complaint Questionnaire and assessing mental health, with particular attention to depression and anxiety, conditions highly prevalent in CKD patients undergoing HD [61,62]. Mental health is particularly relevant in older adults, as depression and anxiety are considered “geriatric giants” [63,64,65]. These conditions should be systematically assessed using tailored instruments, such as the Geriatric Depression Scale, developed for geriatric populations with a simple yes/no format, and the Geriatric Anxiety Inventory, an agree/disagree scale designed to assess late-life anxiety; both instruments place limited emphasis on physical symptoms that may overlap with aging or medical comorbidity [66,67,68].

In clinical practice, older adults with CKD undergoing HD may benefit from a comprehensive cognitive assessment (Table 1), provided that, whenever available, test versions and normative data adjusted for age and education are used, as these factors can affect test scores and their clinical interpretation [69,70]. Sensory impairment, including hearing and visual deficits, as well as increased fatigability, should also be considered on a case-by-case basis. However, practical barriers—including time constraints, costs, staffing shortages, and patient burden—may limit the implementation of a complete test battery. In such cases, more feasible, targeted screening tools may offer a pragmatic approach to identifying cognitive deficits. Attention should be given to tools validated in this population, with the MoCA emerging as a preferred option [71]. Nonetheless, MoCA scores should always be interpreted in light of cultural background and mood symptoms, which may further affect performance [72,73]. For evaluating subjective cognitive complaints, the Kidney Disease Quality of Life Cognitive Function questionnaire, recently revised by Chan et al. [58], may be particularly useful due to its wide availability, ease of use, and minimal training requirements. Additionally, the Dialysis Dementia Risk Score has been proposed as a specific index for HD patients to identify those at higher risk of dementia, facilitating targeted neurological evaluation [74]. Further studies specifically designed for older patients with CKD, including those receiving HD, are needed to determine whether additional disease- or dialysis-related factors should be incorporated into the interpretation of cognitive screening results.

## 4. Gut Dysbiosis in Aging and in CKD

**Figure 3:**
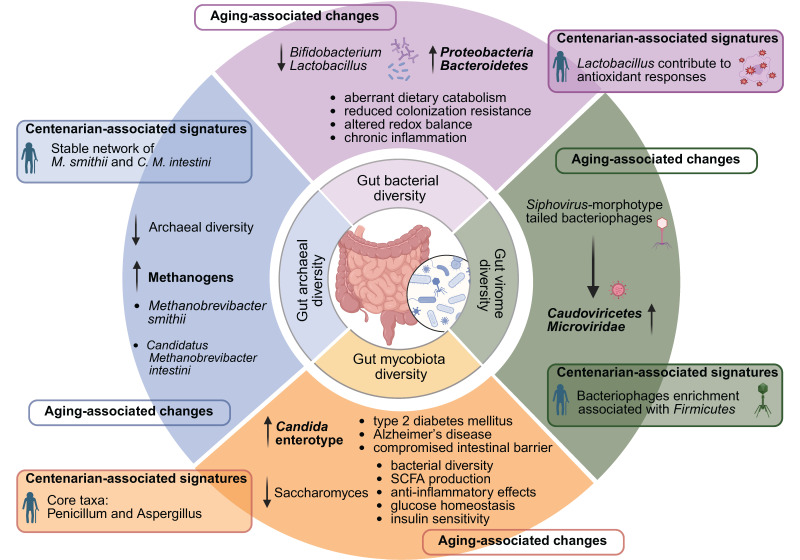
Age-associated changes in the gut microbiota. Aging is characterized by compositional and functional alterations across all microbial components of the gut ecosystem, including bacteria [75], viruses [76,77,78], fungi [79,80], and archaea [81,82]. Centenarians exhibit distinct microbial configurations that may reflect putative adaptive or resilience-related signatures, including taxa potentially involved in antioxidant responses [83], characteristic virome signatures [78], specific fungal profiles [84], and stable archaeal networks linked to microbiome resilience [85]. These findings indicate that aging is associated not only with shifts in microbial composition and function but also with adaptive remodeling of the gut ecosystem, whereas centenarian-associated profiles may reflect selective microbial features linked to exceptional longevity. SCFAs, short-chain fatty acids. Created using BioRender.

The gut is a complex ecosystem inhabited by diverse microbial communities, including bacteria, viruses, fungi, and archaea, which play essential roles in digestion, metabolism, immune regulation, and overall host health. With aging, the gut microbiota undergoes numerous changes, which may contribute to the development of age-related diseases (Figure 3).

The strength of data linking different components of the gut ecosystem to CKD-related cognitive vulnerability in older adults is uneven. Bacterial dysbiosis is supported by more extensive CKD-specific findings, whereas data on the gut virome, mycobiota, and archaeome remain more limited and largely exploratory. Accordingly, the microbiota–UTs–inflammation–BBB framework is used here as a convergent mechanistic model, rather than as a verified linear causal sequence leading to cognitive impairment in older adults with CKD. Findings from non-CKD neurological populations and experimental models are therefore discussed as biological support for pathway plausibility, not as direct proof of CKD-related cognitive decline.

### 4.1. Gut Bacterial Community

The bacterial component of the gut microbiota in CKD patients has been extensively studied [86,87,88]. Here, we focus on alterations potentially relevant to cognitive and neurological outcomes, providing key examples without aiming for completeness.

A prominent mechanism in CKD is the accumulation of gut-derived UTs driven by dysbiosis. CKD is associated with an increased abundance of microbial taxa—such as Enterobacteriaceae, Pseudomonadaceae, and some Clostridiaceae—that possess urease, uricase, and indole/*p*-cresol–forming enzymes. While urease and uricase contribute indirectly by modifying the intestinal environment, indole/*p*-cresol–forming enzymes directly generate precursors that are subsequently converted into protein-bound UTs [89,90,91]. Laiola et al. [92] reported that CKD was associated with both higher circulating UT levels and a distinct serum UT profile, and that metagenomic species enriched in patients with severe CKD were positively correlated with indole- and phenol-derived UTs. Aging itself also elevates protein-bound UTs, including indoxyl sulfate and *p*-cresyl sulfate [93], potentially increasing the vulnerability of older adults with CKD.

Microbial urease hydrolyzes urea to ammonia, which can form ammonium hydroxide in aqueous environments. This increases gut luminal pH, disrupts tight junction proteins, impairs intestinal barrier integrity, and promotes systemic inflammation [94]. This may facilitate UT translocation into the circulation. Animal studies have reported that indoxyl sulfate accumulates in the brain, altering behavior and neurotransmitter levels [95], disrupting circadian rhythms [96], increasing seizure susceptibility [97], and impairing glial function via oxidative and inflammatory pathways [98].

In addition, uremia in CKD reshapes the gut microbiota, reducing beneficial bacterial families such as Prevotellaceae, Lactobacillaceae, and Bifidobacteriaceae [99,100]. These taxa produce short-chain fatty acids (SCFAs), such as butyrate, propionate, and acetate, which support brain homeostasis by reducing neuroinflammation and microglial activation [101], preserving BBB integrity [102], and regulating neurotransmitters and neurotrophic factors [103]. Lu and colleagues [104] identified distinct gut bacterial taxa linked to both CKD and Alzheimer’s disease, suggesting convergent microbial alterations across renal and neurodegenerative phenotypes in later life.

### 4.2. Non-Bacterial Components of the Gut Ecosystem: Virome, Mycobiota, and Archaeome

Bacteriophages can shape bacterial communities through lytic activity, lysogeny, and horizontal gene transfer [105], and gut virome alterations have been reported in mild cognitive impairment, Alzheimer’s disease, and Parkinson’s disease [106,107,108]. However, these findings derive from non-CKD neurological contexts, and no study has directly tested whether virome alterations contribute to cognitive impairment in CKD. The first CKD-associated gut virome signatures were recently described by Zhang et al. [109], who reported enrichment of Siphovirus-morphotype tailed bacteriophages, *Microviridae*, and “*Flandersviridae*”, frequently linked to potentially pathogenic bacteria implicated in inflammation and UT production. Functional analyses suggested a shift toward lytic replication, potentially increasing the release of proinflammatory bacterial components and influencing mucosal and innate immune responses [109]. These findings suggest possible convergence with pathways relevant to brain vulnerability, including UT generation and inflammation, but remain indirect and should be interpreted within the broader context of aging-related gut virome remodeling. In addition, the large fraction of unclassified viral sequences in human gut virome studies (“viral dark matter”) may obscure disease-relevant viral functions and host–microbiota interactions [110].

CKD-related alterations of the gut mycobiota have also been reported, although their clinical relevance remains uncertain. In healthy individuals, stable fungal colonizers are mainly represented by *Candida* spp. and Dipodascaceae, whereas many other fungi may reflect transient dietary or environmental exposure [111]. In CKD, shifts involving *Saccharomyces*, *Candida*, *Bjerkandera*, *Rhodotorula*, *Ganoderma*, *Apiotrichum*, *Cystobasidium*, and *Meyerozyma* have been associated with inflammatory and immune-activation markers [112,113]. In ESKD, reduced *Saccharomyces cerevisiae* and enrichment of opportunistic fungi have been linked to creatinine, homocysteine, and phenylacetylglycine, suggesting a relationship with uremic metabolic imbalance [114]. Although mycotoxins may affect neurological disease through BBB disruption, oxidative stress, neuroinflammation, and amyloid-β-related pathways [115], and gut mycobiome alterations have been associated with mild cognitive impairment and Alzheimer’s disease biomarkers [116], these observations are not CKD-specific. Conversely, in Parkinson’s disease, gut fungal load appears to be driven mainly by aging rather than disease status [117], underscoring the uncertainty of disease-specific mycobiota effects.

Evidence on the gut archaeome is even more limited. In HD patients, *Methanosphaera* has been positively correlated with IL-1β expression [118], whereas *Methanobrevibacter* abundance has been negatively correlated with plasma trimethylamine *N*-oxide levels [119]. Non-CKD studies provide conflicting links with cognition: *Methanobrevibacter smithii* has been associated with better executive function, inhibitory control, cognitive flexibility, attention, and working memory, with supportive fecal microbiota transplantation (FMT) data in mice [120]; however, *M. smithii* enrichment has also been reported in Parkinson’s disease [121] and in older adults with severe cognitive impairment, together with increased methanogenesis and reduced SCFA- and neurotransmitter-related pathways [122].

Thus, current evidence does not support a direct role for the virome, mycobiota, or archaeome in CKD-related cognitive impairment. These components should be considered emerging dimensions of multi-kingdom dysbiosis that may intersect with inflammatory, metabolic, or UT-related pathways, rather than verified mechanistic targets for cognitive decline in older adults with CKD.

## 5. Dietary Recommendations in CKD: The Possible Contribution of the Microbiota

**Figure 4:**
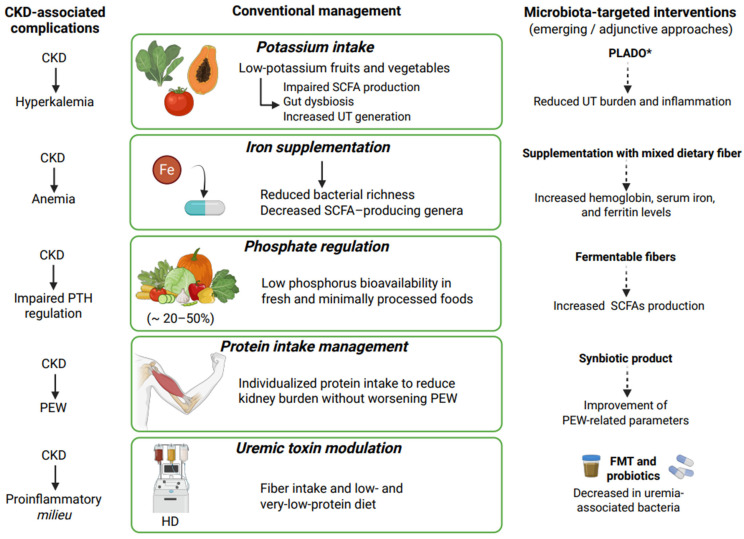
Overview of conventional management and microbiota-targeted interventions in chronic kidney disease (CKD). CKD-related complications (**left**) are addressed through standard strategies (**center**), including dietary, pharmacological, and dialysis-based approaches. These conventional strategies represent guideline-based or clinically established components of CKD care. Emerging microbiota-directed interventions (**right**), such as the plant-dominant low-protein diet (PLADO), synbiotics, fermentable fibers, and fecal microbiota transplantation (FMT), are presented as possible complementary approaches supported by evolving evidence. However, they are not yet established as routine CKD care. * PLADO is consistent with current dietary approaches for selected metabolically stable patients with CKD G3–G5 not receiving dialysis; however, its specific microbiota-mediated effects remain investigational. Solid arrows indicate CKD-related pathological processes and potential adverse effects of conventional management, whereas dashed arrows indicate potential microbiota-mediated improvement of these conditions. PTH, parathyroid hormone; PEW, protein-energy wasting; SCFAs, short-chain fatty acids; UTs, uremic toxins; HD, hemodialysis. Created using BioRender.

Patients with CKD, particularly those undergoing HD, often require tailored nutritional protocols and specific supplementation to address disease-related imbalances. Within this context, dietary strategies that modulate the gut microbiota may be especially relevant, as they could support CKD management while also potentially influencing pathways linked to neurological vulnerability (Figure 4). Because nutritional priorities differ substantially across CKD stages, dialysis status, hyperkalemia or phosphate risk, and nutritional vulnerability, the following sections stratify the discussion by CKD G3–G5 not receiving dialysis, CKD G5HD, older adults, and patients at risk of PEW whenever the evidence allows. In older HD patients, dietary planning should also account for frailty, polypharmacy, gastrointestinal symptoms, poor appetite, adherence, and malnutrition risk, as these factors may influence both microbiota composition and the feasibility of microbiota-oriented dietary strategies.

### 5.1. Potassium Balance

Declining kidney function increases the risk of hyperkalemia, which is associated with CKD progression [123], adverse cardiovascular outcomes [124], and higher mortality [125,126]. Potassium imbalance can impair neuronal excitability [127,128] and has been associated with cognitive deficits [129], suggesting that its control may be important for both limiting CKD-related complications and preserving neuronal function and cognitive performance.

Dietary management in CKD has traditionally included limiting potassium-rich fruits and vegetables; however, this strategy may reduce fermentable fiber intake, with potential downstream effects on SCFA production, gut microbiota composition, and UT generation [130]—pathways that may also be relevant to neuroinflammation. Current KDIGO (Kidney Disease: Improving Global Outcomes) recommendations for CKD G3–G5 not receiving dialysis emphasize that potassium restriction should be individualized, particularly in patients with current or recurrent hyperkalemia, and should focus on highly processed foods and other sources of readily absorbable potassium, such as processed meats, dairy products, fruit juices, and salt substitutes containing potassium chloride [2]. Rather than broadly restricting plant foods, prioritizing minimally processed sources may represent a more microbiota-compatible approach, as their alkalinizing properties, carbohydrate content, and fiber may favor intracellular potassium shifts and enhance fecal potassium excretion. Nevertheless, this evidence derives mainly from physiological and observational data in CKD G5HD populations and should not be interpreted as support for unrestricted potassium intake [131].

Plant-based proteins can also meet both quantitative and qualitative protein requirements without increasing the risk of deficiencies compared with animal-based proteins, provided that intake is adequate and the diet includes varied plant sources [132]. Recent clinical evidence further supports a more individualized approach to potassium management. In a 6-week feasibility trial, Avesani et al. [133] evaluated a plant-based diet in hyperkalemia-prone patients with CKD G4–G5 not receiving dialysis who were receiving sodium zirconium cyclosilicate. The intervention improved dietary quality and increased the intake of healthy plant foods, while plasma potassium remained stable within the normal range for most patients. These findings suggest that, in selected and closely monitored patients, potassium binders may help enable a more liberal plant-based dietary pattern; however, this strategy should still be considered individualized and not generalized to all patients with CKD.

Accordingly, a plant-dominant low-protein diet (PLADO) may be considered in metabolically stable patients with CKD G3–G5 not receiving dialysis, providing 0.6–0.8 g/kg/day protein with ≥50% from plant sources. This approach may reduce UT burden and systemic inflammation while favorably modulating the gut microbiota by promoting saccharolytic metabolism, supporting SCFA generation, and reducing gut dysbiosis [134,135], with reported reductions in indoxyl sulfate and *p*-cresyl sulfate levels [135]. Within this approach, potassium intake can be managed by selecting low-potassium fruits (e.g., apples, pears, grapes, and berries) and vegetables (e.g., zucchini, lettuce, cucumbers, and green beans), as well as by using cooking methods such as boiling, soaking, or leaching [135]. Notably, the shift toward plant-derived protein sources in CKD management is consistent with broader dietary patterns associated with brain health: plant-based dietary patterns, including Mediterranean and MIND diets [136,137], have been associated with better cognitive outcomes and reduced dementia risk. This convergence supports a shared nutritional rationale across kidney, gut microbiota, and brain health, although direct evidence that these dietary patterns improve cognitive outcomes in CKD remains limited.

### 5.2. Iron Supplementation

Anemia is a common complication of CKD, particularly in advanced stages, typically driven by reduced renal erythropoietin production and iron deficiency resulting from blood loss, nutritional deficits, impaired absorption, elevated hepcidin, and increased erythropoiesis-stimulating agent–driven iron utilization; as such, it represents both a major clinical determinant and a therapeutic target [138].

Anemia is associated with an increased risk of cognitive decline and dementia, potentially through mechanisms involving chronic cerebral hypoxia, oxidative stress, and neuroinflammation, which contribute to neuronal damage and reduced brain resilience [139]. A neuroimaging study in patients with chronic anemia showed that lower hemoglobin levels correlate with diffuse reductions in white matter volume, particularly in watershed regions, consistent with chronic hypoxic–ischemic injury [140]. Recognition and treatment of anemia may therefore be relevant to CKD-related brain vulnerability, although direct evidence linking anemia correction to improved brain outcomes in CKD remains limited.

Iron supplementation is also essential to prevent fatigue, reduced exercise capacity, tissue hypoxia, increased cardiovascular risk, and impaired quality of life [141,142]; however, supplementation strategies must be selected according to dialysis status, iron indices, inflammatory burden, and route of administration. Liu and colleagues [143] showed that in anemic CKD G5HD patients, oral iron reduced bacterial richness and decreased the abundance of key SCFA-producing genera, including *Blautia*, *Coprococcus*, and the family Lachnospiraceae, whereas intravenous iron was associated with greater bacterial richness and higher relative abundance of taxa such as *Akkermansia* and *Ruminococcus*. Notably, *Lactobacillus* was more abundant after oral iron, underscoring that route-dependent microbiota changes are not uniformly deleterious or beneficial.

In healthy middle-aged women, high-dose oral iron supplementation (>100 mg/day) was associated with dose-dependent changes in gut microbial composition, including increased Proteobacteria and reduced *Akkermansia*, *Butyricicoccus*, *Ruminococcus*, and *Faecalibacterium* [144]. Although not CKD-specific, these findings support the biological plausibility that excess luminal iron may affect taxa involved in gut barrier function, microbial cross-feeding, and SCFA production. Together with evidence from CKD G5HD patients, these data support considering the route of administration when evaluating the intestinal effects of iron therapy [145].

Beyond pharmacological iron supplementation, microbiota-targeted dietary interventions may contribute to improved hematological parameters. In a randomized placebo-controlled trial involving 162 patients with CKD G5HD, supplementation with mixed dietary fiber (galactomannan, resistant dextrin, fructooligosaccharides, and starch) increased hemoglobin, serum iron, and ferritin levels and was accompanied by enrichment of *Bifidobacterium adolescentis*, *Lactobacillus*, and Lactobacillaceae, together with higher circulating butyrate concentrations [146]. Because the observed associations between microbial changes, butyrate levels, and hematological parameters were correlational, the underlying mechanisms remain to be established.

### 5.3. Phosphate Regulation

As kidney function declines, phosphate retention contributes to increased parathyroid hormone (PTH) secretion and broader disturbances in mineral metabolism, promoting renal osteodystrophy, mineral and bone disorder (MBD), vascular calcification, and increased cardiovascular morbidity and mortality [147]. Phosphate dysregulation may also be relevant to brain vulnerability in CKD. Higher serum phosphorus levels have been associated with increased risk of incident dementia in a large non-dialysis cohort [148]. Consistently, preclinical evidence suggests that CKD-related disturbances in mineral metabolism may affect brain function: in an adenine-induced rat model of CKD-associated hyperphosphatemia, renal dysfunction was associated with impaired cognitive performance and reduced cortical Klotho expression [149].

Phosphorus is widely present in commonly consumed foods such as dairy products, meat, fish, legumes, nuts, and whole grains; however, not all sources have the same physiological impact. In patients with CKD, fresh and minimally processed foods—particularly plant-based sources—are generally favored due to their lower phosphorus bioavailability (generally <50%, and approximately 20–30% for phytate-bound phosphorus), compared with animal-based sources (~60–70%) and especially inorganic phosphate additives in processed foods (~80–100%), which are the most readily absorbed. Accordingly, foods containing phosphate additives (e.g., processed cheese, cured meats, baked goods, and soft drinks) should be avoided, while animal-based phosphorus-rich foods should be consumed in moderation [150].

Beyond dietary phosphate restriction, complementary nutritional strategies based on fermentable prebiotic fibers may contribute to phosphate homeostasis in CKD, although current evidence remains limited and partly derived from preclinical models. In a rat model of CKD-MBD, dietary supplementation with 10% inulin reduced plasma phosphate and PTH levels and was associated with marked remodeling of the cecal microbiota, including increased *Bifidobacterium*, *Allobaculum*, and unclassified Lachnospiraceae, together with reduced *Lactobacillus*, *Oscillospira*, *Adlercreutzia*, *Dorea*, and unclassified Clostridiaceae and Ruminococcaceae [151]. Clinical evidence supporting a direct phosphate-lowering effect of inulin in CKD patients is still limited; however, in a small 6-month study of patients with CKD G3–G4 not receiving dialysis, inulin added to a low-protein diet modulated gut microbiota composition, with increased Bifidobacteriaceae and reduced Enterobacteriaceae, and was accompanied by reductions in inflammatory and oxidative stress markers [152]. Resistant starch represents another fermentable fiber of interest, as it escapes digestion in the small intestine and undergoes microbial fermentation in the colon, promoting SCFA production and modulating gut microbial composition. Recent meta-analyses of randomized trials suggest that resistant starch supplementation may modestly reduce serum phosphate and improve selected renal function or UT-related indices in CKD, although effects on inflammatory markers are inconsistent and the underlying mechanisms remain incompletely defined [153,154]. These findings support resistant starch as a promising microbiota-directed dietary strategy, but its clinical role is not yet established as routine CKD care.

Additional preclinical work has explored gut-targeting approaches that combine intestinal phosphate binding with microbiota modulation. In an adenine-induced rat model of CKD-associated hyperphosphatemia, a biomimetic chitosan-based prebiotic microsphere (CSM@5) reduced serum phosphate levels and increased fecal phosphate excretion, while partially restoring gut microbiota composition toward a profile resembling healthy controls [155].

### 5.4. Protein Intake and Protein-Energy Wasting

Protein-energy wasting (PEW) is characterized by depletion of body protein and energy reserves, typically reflected by loss of muscle and/or fat mass [156]. Its prevalence increases with CKD severity, ranging from 11% to 54% in CKD G3–G5 not receiving dialysis and from 28% to 54% (25th–75th percentiles) among patients on maintenance dialysis [157].

In a study of 102 patients with ESKD receiving dialysis, stratified by dialysis modality and PEW status, PEW was associated with a lower abundance of selected SCFA-producing genera, including *Roseburia*, *Phascolarctobacterium*, and *Blautia*, together with a higher abundance of potentially pathogenic taxa such as *Escherichia*. These associations varied by dialysis modality and were accompanied by higher inflammatory markers and predicted enrichment of branched-chain amino acid degradation pathways [158]. In a longitudinal study of patients on maintenance HD, lower baseline abundance of Actinobacteria and Bifidobacteriaceae was associated with subsequent reductions in lean tissue mass and increased risk of PEW development over 1 year [159].

In patients with CKD not receiving dialysis, low-protein diets are often prescribed both to slow kidney function decline, as supported by observational evidence linking adherence to a low-protein diet with slower eGFR decline [160], and to reduce glomerular hyperfiltration and metabolic complications such as acidosis and phosphate retention [161]. However, a meta-analysis showed that low-protein diets in non-diabetic adults with CKD G3 not receiving dialysis have little or no effect on mortality or progression to ESKD, whereas very-low-protein diets in CKD G4–G5 not receiving dialysis likely reduce ESKD risk. These diets appear generally safe regarding PEW, with few cases of malnutrition reported, although data on body weight, nutritional status, and quality of life remain limited [162]. After the transition to maintenance HD, nutritional management becomes more complex, as protein intake generally needs to increase to offset dialysis-related amino acid losses and the catabolic effects of inflammation, metabolic acidosis, and the HD procedure itself [163]. In this setting, adequate protein provision should be combined with fermentable fiber intake and careful consideration of protein quality and source, including the potential role of plant-derived proteins discussed below. This approach is clinically relevant because a higher dietary protein-to-fiber ratio has been associated with higher circulating concentrations of protein-bound UTs in anuric HD patients, and higher protein intake may increase colon-derived UT generation when not balanced by fermentable substrates [164,165].

Protein source should therefore be interpreted within the broader dietary matrix. Plant-predominant diets are generally associated with higher fecal SCFA levels, likely due to their greater content of fermentable fiber. In contrast, animal-based dietary patterns have been associated with enrichment of bile-tolerant taxa, including *Alistipes*, *Bilophila*, and *Bacteroides*, reduced abundance of plant-polysaccharide-degrading Firmicutes, and a shift from carbohydrate toward amino acid fermentation [166,167]. This balance is particularly important in older adults with CKD, in whom dietary protein restriction must be weighed against sarcopenia risk. Hung et al. [168] reported that, in older patients with CKD G3b–G5 not receiving dialysis, a low-protein diet (≤0.8 g/kg/day) was not associated with deterioration of serum albumin or appendicular skeletal muscle mass index over 1 year. However, in older adults with CKD G3–G5 not receiving dialysis, adding a 6% low-protein formula providing energy, limited protein, fatty acids, and micronutrients to a low-protein diet was associated with more favorable changes in handgrip strength and gait speed over 3 months, without significant between-group differences in nutritional status or body composition [169].

Although the impact of PEW on cognitive function appears less well established than that of other CKD-related alterations, cross-sectional data from maintenance HD populations suggest that PEW is associated with lower global cognitive scores and poorer performance across selected domains, including executive function, attention, orientation, delayed recall or short-term memory, and language-related abilities [170,171]. In parallel, experimental CKD data show reduced hippocampal excitatory synaptic transmission and a correlation between lower body weight and impaired synaptic measures, supporting a plausible link between nutritional-metabolic status and brain vulnerability rather than a proven causal pathway [171].

Together, these findings support considering PEW as a potential modifier of brain vulnerability in advanced CKD, particularly in patients receiving maintenance HD, although further studies are needed to clarify its relationship with specific cognitive outcomes.

### 5.5. Uremic Toxins

As discussed above, the accumulation of UTs in CKD represents a key pathophysiological driver of disease progression and may contribute to brain dysfunction through mechanisms including neuroinflammation, oxidative stress, and disruption of BBB integrity. However, their management remains challenging, as conventional approaches such as dialysis are largely ineffective in removing protein-bound toxins and do not address their intestinal generation. Beyond indoxyl sulfate and *p*-cresyl sulfate, other uremic solutes and neuroactive metabolites may also be relevant to cognitive vulnerability. Kynurenic acid, a tryptophan–kynurenine pathway metabolite, has been identified among circulating metabolites associated with early cognitive decline in non-CKD human metabolomic studies [172], while CKD-related dysbiosis and impaired renal clearance may alter tryptophan–kynurenine metabolism, with increased kynurenine-pathway metabolites reported in experimental CKD settings [16]. Hippuric acid, a diet- and microbiota-related protein-bound UT, has been proposed to influence brain vulnerability indirectly, including through interference with organic anion transporters involved in the handling of other neurotoxic solutes; however, its clinical association with cognitive performance in CKD remains uncertain [173,174].

In a gut-humanized CKD mouse model, oat-derived dietary fibers, including oat-resistant starch and oat β-glucan, increased SCFA levels, improved gut dysbiosis and intestinal barrier function, and reduced CKD-related solutes, including creatinine, indoxyl sulfate, and *p*-cresyl sulfate [175]. In anuric HD patients, a lower protein-to-fiber ratio was also associated with lower circulating concentrations of these protein-bound UTs [163]. These results should be interpreted with caution, as the underlying mechanisms remain incompletely defined in humans and causal relationships, as well as clinical applicability, have yet to be established.

Preliminary data also suggest a beneficial role of low- and very-low-protein dietary regimens. In the MEDIKA2 randomized crossover trial, a very-low-protein diet supplemented with ketoanalogues was associated with a substantial reduction in total and free indoxyl sulfate and *p*-cresyl sulfate in CKD G3b–G4 patients, alongside changes in gut microbiota composition, including reduced abundance of Proteobacteria and increased Bacteroidota, Bifidobacteriaceae, Ruminococcaceae, and selected SCFA-producing taxa. These findings are consistent with a shift toward a less proteolytic and more saccharolytic microbial profile, although the effect appeared to be driven mainly by protein restriction rather than ketoanalogue supplementation alone [176].

Finally, FMT has been explored as an experimental gut-targeting strategy. In preclinical CKD models, FMT from healthy donors reshaped gut microbial composition, restored selected *Lactobacillus* species, modified microbial amino acid metabolism, and reduced the accumulation of protein-bound UTs, including indoxyl sulfate and *p*-cresyl sulfate [177]. However, these effects appear to be model- and pathway-specific: in an adenine-induced CKD mouse model, FMT reduced *p*-cresol–derived toxins, including *p*-cresyl sulfate and *p*-cresyl glucuronide, but had no consistent effect on indole-derived UTs or renal function [178]. Early clinical data further support the feasibility and safety of FMT in CKD patients; however, available evidence remains limited to small trials, microbiota changes appear modest, UT levels have not yet been directly assessed, and effects on kidney or neurological outcomes require confirmation in larger and longer studies [179].

### 5.6. Probiotics, Synbiotics, and Postbiotics

Probiotics (live microorganisms that, when administered in adequate amounts, confer a health benefit on the host) and synbiotics (combinations of probiotics with prebiotic substrates that enhance their survival and metabolic activity) have been proposed as adjunctive strategies to modulate gut dysbiosis in CKD. More broadly, non-CKD experimental *and in vitro* studies indicate that microbiota modulation can increase SCFA production, support intestinal barrier integrity, and attenuate inflammatory signaling, providing general mechanistic background for the microbiota–SCFA–barrier–inflammation axis [180,181]. However, these findings should be interpreted as background biological evidence rather than direct support for cognitive benefit in older adults with CKD or HD.

In CKD animal models, administration of specific bacterial strains such as *Phocaeicola plebeius*—reported as *Bacteroides plebeius* in the original study—and *Faecalibacterium prausnitzii* has been associated with improved gut barrier integrity, lower inflammatory or endotoxin-related markers, and attenuation of skeletal muscle atrophy or PEW-related muscle wasting [182,183]. Similarly, in CKD rat models, synbiotic interventions combining *Bifidobacterium longum* with prebiotic substrates have been associated with reductions in serum phosphate, PTH, and indoxyl sulfate; in one study, these effects were accompanied by improved jejunal ZO-1 expression and shifts in gut microbial composition, suggesting a possible contribution of gut barrier modulation to phosphate handling [184,185].

However, clinical evidence remains limited and inconsistent. Some studies using synbiotic formulations have reported improvements in hematological parameters in HD patients, including hemoglobin, hematocrit, ferritin, transferrin saturation, and red blood cell count in one trial [186], and increased hemoglobin after probiotic or synbiotic supplementation in another trial assessing mental health and quality-of-life outcomes [187]. These effects may be related to reduced inflammatory burden or microbiota modulation, but mechanistic evidence remains indirect. In addition, a synbiotic gel containing *Lactobacillus acidophilus* NCFM, *Bifidobacterium lactis* Bi-07, inulin, omega-3 fatty acids, and vitamins reduced gastrointestinal symptom burden in HD patients and was accompanied by relative preservation of protein intake and lean tissue mass compared with placebo, although nutritional and inflammatory outcomes were not consistently significant, and cannot be attributed specifically to microbiota modulation [188]. In line with this mixed evidence, a recent systematic review and meta-analysis of randomized controlled trials reported that probiotic and synbiotic supplementation in CKD was associated with reductions in blood urea nitrogen and *C*-reactive protein, whereas effects on eGFR and serum creatinine were not significant [189].

In HD patients, synbiotic supplementation did not significantly improve serum phosphate, and one trial reported an increase in PTH within the synbiotic group, although between-group differences were not significant [190]. Evidence in CKD patients not receiving dialysis is more encouraging for UT and inflammatory endpoints: in CKD G3b–G4 patients, synbiotic supplementation reduced indoxyl sulfate and hsCRP and shifted gut microbiota composition [191]. A recent systematic review and network meta-analysis of RCTs in CKD G3–G5 suggests that microbiota-targeting nutritional interventions may reduce selected UTs, with probiotics ranking highest for total and free indoxyl sulfate and prebiotics ranking highest for *p*-cresyl sulfate and urea [192]. However, these comparative estimates should be interpreted cautiously because of small sample sizes, heterogeneous interventions, inconsistent microbiota data, and limited evidence regarding major clinical outcomes, safety in frail HD populations, and direct effects on specific cognitive domains.

Postbiotics, defined as preparations of inanimate microorganisms and/or their components, may represent an additional microbiota-derived strategy in CKD, although current evidence is limited to conceptual and preclinical work in kidney-related conditions, with no human CKD studies and no data on cognitive outcomes in older adults with CKD [193].

Overall, current evidence supports further investigation of probiotics, synbiotics, and postbiotics in CKD, but does not support their routine use for improving hard clinical or neurocognitive outcomes.

### 5.7. Clinical Implications and Practical Considerations

The nutritional approaches discussed above may influence microbiota-related metabolic and inflammatory pathways in CKD, but their feasibility and safety in older adults require individualized assessment. Current CKD nutrition guidelines emphasize that dietary strategies should be tailored to kidney function, dialysis status, nutritional risk, comorbidity burden, and biochemical safety parameters, including markers of electrolyte, phosphate, acid–base, and PEW-related imbalance [194]. This is particularly relevant in geriatric CKD patients, in whom frailty, sarcopenia, multimorbidity, and reduced nutritional reserves may limit the applicability of restrictive dietary approaches and increase the risk of functional decline [195].

In practical terms, referral to a renal dietitian should be prioritized when older CKD or HD patients show nutritional vulnerability, frailty or sarcopenia, poor intake or adherence difficulties, recurrent potassium or phosphate imbalance, gastrointestinal intolerance, or when substantial plant-based or microbiota-oriented dietary changes are being considered. Accordingly, microbiota-oriented dietary strategies should not be applied as standardized prescriptions. They should be implemented under clinical supervision, preferably involving nephrologists and renal dietitians, with monitoring of dietary intake, body weight trajectory, nutritional status, serum electrolytes, phosphate, bicarbonate, gastrointestinal tolerance, adherence, and functional or cognitive changes when clinically indicated. In this context, microbiota profiling and measurement of gut-derived UTs should currently be regarded mainly as research tools, as they are not yet sufficiently standardized to inform routine care [196]. Recent feasibility data suggest that plant-based dietary patterns may be implementable even in selected hyperkalemia-prone CKD patients when combined with potassium-binder therapy [133]. Similarly, prebiotic, probiotic, and synbiotic interventions have been investigated as microbiota-targeted strategies in CKD, but their effects appear heterogeneous and their tolerability, strain- or substrate-specific effects, and long-term safety remain insufficiently established, particularly in frail, multimorbid, or immunocompromised older adults [197].

## 6. Conclusions and Future Directions

Older adults with CKD, particularly those undergoing HD, represent a clinically vulnerable population in whom cognitive impairment is frequent yet underrecognized. However, structured cognitive assessment remains limited and lacks population-specific adaptation. The tools discussed in this review should therefore be interpreted as a clinically reasoned framework for early detection, rather than as components of a fully validated standard approach.

Gut dysbiosis may represent a plausible mediator of kidney–brain interactions, although the strength of evidence varies across microbial components. While the bacterial fraction is supported by more consistent mechanistic and clinical data, evidence on the gut virome, mycobiota, and archaeome remains limited and largely descriptive. Integrated multi-kingdom analyses evaluating interactions among the virome, bacteriome, mycobiome, and archaeome are still lacking, limiting a systems-level understanding of dysbiosis in CKD.

Dietary strategies that increase fermentable fiber intake and emphasize minimally processed plant-derived foods may contribute to kidney and metabolic health while shaping gut microbiota–mediated pathways potentially relevant to brain vulnerability. In this context, microbiota-targeted interventions, including prebiotics, synbiotics, and FMT, represent potential strategies in CKD; however, current evidence is derived primarily from preclinical studies and small clinical trials. Routine clinical use cannot yet be recommended, as available data rely largely on surrogate endpoints, including SCFA production, UT levels, and inflammatory markers, rather than clinically relevant outcomes, such as cognitive decline. Moreover, substantial heterogeneity in study design, intervention type, duration, and patient populations limits cross-study comparisons and the ability to draw firm conclusions or to identify optimal strategies across specific CKD or HD subgroups.

Establishing whether modulation of the gut microbiota can translate into clinically meaningful cognitive benefit remains a key unmet challenge. Future longitudinal and interventional studies in well-characterized older CKD and HD cohorts should integrate microbiota profiling, UT measurements, inflammatory markers, detailed nutritional assessment, and domain-specific cognitive endpoints, because current evidence does not yet allow microbiota- or nutrition-related mechanisms, including UT accumulation and SCFA alterations, to be mapped onto specific cognitive domains. In this context, early microbiota-targeted nutritional strategies should also be evaluated for their potential to prevent or delay cognitive decline.

## Figures and Tables

**Table 1:** Proposed battery of cognitive and mood assessment tools for older adults with chronic kidney disease undergoing hemodialysis.

Key Domains Assessed	Test	Cognitive Functions/Outcome	Feasibility Considerations
Global cognition	MMSE	Global cognition	Brief and widely used, but limited sensitivity to executive dysfunction and milder cognitive impairment; scores should be interpreted according to age and education
	Modified MMSE	Orientation, memory, attention, language, visuospatial skills	More informative than the standard MMSE but still limited for executive dysfunction; requires attention to education, culture, sensory impairment, and fatigue
	MoCA	Executive function, memory, attention, language, visuospatial skills	Relatively feasible and more sensitive to executive and multidomain impairment; interpretation should consider education, culture, mood symptoms, sensory impairment, and fatigue
Memory/executive function	Mini-Cog Test	Memory and executive function screening	Very brief and easy to administer; useful for rapid screening but limited in domain-specific characterization
Attention/executive functions	Trail Making Test Part B	Attention/executive functions	Sensitive to executive dysfunction but influenced by visual ability, motor speed, and education
	Digit Symbol-Coding Test	Executive function, attention, information processing	Informative for processing speed and attention but requires visual scanning, motor speed, and sustained effort
Long-term verbal memory	Rey Auditory Verbal Learning Test—immediate and delayed recall	Learning and memory	Provides detailed memory assessment but is more time-consuming and may be burdensome in frail or fatigued patients
Complex attention	Symbol Digit Modalities Test	Sustained attention and processing speed	Sensitive to processing speed and attention; performance may be affected by visual-motor limitations and fatigue
	Digit Span Forward	Attentional capacity	Brief and low-burden; useful for attentional capacity but limited as a standalone cognitive assessment
Executive function	Digit Span Backward	Working memory	Brief but cognitively demanding; useful for working memory and executive control, but affected by fatigue and reduced attention
	F-A-S Phonemic Fluency	Cognitive organization, initiation, execution of search strategies	Useful for executive-language assessment; performance is influenced by education, language, and cultural background
Language	RBANS Semantic Fluency	Semantic fluency screening	Provides domain-specific information but requires trained administration and interpretation; language and education should be considered
	RBANS Picture Naming	Naming function	Useful for naming assessment but may be affected by visual impairment, cultural familiarity with stimuli, and education
Visuospatial/perceptual-motor functions	RBANS Line Orientation	Visuospatial orientation	Informative for visuospatial function but dependent on visual acuity and patient cooperation
	RBANS Figure Copy	Organizational and visuoconstructional abilities	Useful for visuoconstructional assessment but influenced by visual ability, motor function, and fatigue
Depression and anxiety	GDS	Depression screening	Feasible in older adults; reduced emphasis on somatic symptoms supports use in medically complex patients
	GAI	Anxiety screening	Feasible in older adults; simple response format and limited somatic emphasis support use in medically complex patients
Subjective memory complaints	MAC-Q	Self-reported memory complaints	Low-burden tool for subjective complaints; should complement, not replace, objective cognitive assessment

Practical considerations are based on administration burden, need for trained personnel, sensory or motor requirements, fatigue, and interpretive limitations. They are intended to support clinical implementation and should not be interpreted as formally validated feasibility scores. MMSE, Mini-Mental State Examination; MoCA, Montreal Cognitive Assessment; RBANS, Repeatable Battery for the Assessment of Neuropsychological Status; GDS, Geriatric Depression Scale; GAI, Geriatric Anxiety Inventory; MAC-Q, Memory Complaint Questionnaire.

## Data Availability

No new data were created or analyzed in this study. Data sharing is not applicable to this article.

## Abbreviations

The following abbreviations are used in this manuscript:

CKD	Chronic Kidney Disease
HD	Hemodialysis
GFR	Glomerular Filtration Rate
KDIGO	Kidney Disease: Improving Global Outcomes
UTs	Uremic Toxins
BBB	Blood–Brain Barrier
ESKD	End-Stage Kidney Disease
MMSE	Mini-Mental State Examination
RBANS	Repeatable Battery for the Assessment of Neuropsychological Status
MoCA	Montreal Cognitive Assessment
GDS	Geriatric Depression Scale
GAI	Geriatric Anxiety Inventory
MAC-Q	Memory Complaint Questionnaire
SCFAs	Short-Chain Fatty Acids
IL-18	Interleukin-18
TNF-α	Tumor Necrosis Factor alpha
IFN-γ	Interferon gamma
IL-2Rα	Interleukin-2 Receptor alpha
IL-9	Interleukin-9
TNF-β	Tumor Necrosis Factor beta
PLADO	Plant-Dominant Low-Protein Diet
FMT	Fecal Microbiota Transplantation
PEW	Protein-Energy Wasting
PTH	Parathyroid Hormone
MIND	Mediterranean-DASH Intervention for Neurodegenerative Delay
MBD	Mineral and Bone Disorder

## References

[1] Herrington W.G., Judge P.K., Grams M.E., Wanner C. (2025). Chronic kidney disease. Lancet.

[2] Stevens P.E., Ahmed S.B., Carrero J.J., Foster B., Francis A., Hall R.K., Herrington W.G., Hill G., Inker L.A., Kazancıoğlu R. (2024). KDIGO 2024 Clinical Practice Guideline for the Evaluation and Management of Chronic Kidney Disease. Kidney Int..

[3] World Health Organization (WHO) Reducing the Burden of Noncommunicable Diseases Through Promotion of Kidney Health and Strengthening Prevention and Control of Kidney Disease. https://apps.who.int/gb/ebwha/pdf_files/EB156/B156_CONF6-en.pdf.

[4] GBD 2023 Chronic Kidney Disease Collaborators (2025). Global, regional, and national burden of chronic kidney disease in adults, 1990–2023, and its attributable risk factors: A systematic analysis for the Global Burden of Disease Study 2023. Lancet.

[5] Centers for Disease Control and Prevention (CDC) (2023). Chronic Kidney Disease in the United States.

[6] Nitta K., Okada K., Yanai M., Takahashi S. (2013). Aging and chronic kidney disease. Kidney Blood Press. Res..

[7] Shahbaz H., Rout P., Gupta M. (2024). Creatinine clearance. StatPearls [Internet].

[8] Dowling T.C., Wang E.S., Ferrucci L., Sorkin J.D. (2013). Glomerular filtration rate equations overestimate creatinine clearance in older individuals enrolled in the Baltimore Longitudinal Study on Aging: Impact on renal drug dosing. Pharmacotherapy.

[9] Wang Y., Liu J., Fang Y., Zhou S., Liu X., Li Z. (2024). Estimating the global prevalence of secondary hyperparathyroidism in patients with chronic kidney disease. Front. Endocrinol..

[10] Virzì G.M., Clementi A., Ronco C., Zanella M. (2025). Red cell death in renal disease: The role of eryptosis in CKD and dialysis patients. Cells.

[11] Kourtidou C., Tzionalos K. (2023). Epidemiology and Risk Factors for Stroke in Chronic Kidney Disease: A Narrative Review. Biomedicines.

[12] Roy A., Roy R., Bhattacharya P., Borah A. (2025). The vicious consequences of chronic kidney disease on cognitive impairment and Alzheimer’s disease. ACS Chem. Neurosci..

[13] Huang Z., Yaffe K., Li C., Xiao C., Pan Y., Sun X., Anderson A.H., He J., Jaar B.G., Han H. (2026). Chronic kidney disease severity and risk of cognitive impairment. JAMA Netw. Open.

[14] Chen Y., Zhu R., Zhu X., Zhu Q., Luo Z., Mu J., Zhang M., Ma S. (2026). Bibliometric analysis of cognitive impairment secondary to chronic kidney disease. Medicine.

[15] Mendoza-Ruvalcaba N.M., Vázquez-Núñez K.P., Rodríguez-Díaz M. (2026). The role of cognitive reserve on successful aging in community-dwelling older adults. Appl. Neuropsychol. Adult.

[16] Wagner C.A., Frey-Wagner I., Ortiz A., Unwin R., Liabeuf S., Suzumoto Y., Iervolino A., Stasi A., Di Marzo V., Gesualdo L. (2025). The role of the intestinal microbiome in cognitive decline in patients with kidney disease. Nephrol. Dial. Transplant..

[17] Coradduzza D., Sedda S., Cruciani S., De Miglio M.R., Ventura C., Nivoli A., Maioli M. (2023). Age-related cognitive decline, focus on microbiome: A systematic review and meta-analysis. Int. J. Mol. Sci..

[18] Zhou L., Wang Z., Wang M., Li X., Ren Q. (2025). Gut microbiome mediates the causal relationship between chronic kidney disease and cognitive dysfunction: A Mendelian randomization study. Physiol. Behav..

[19] Raskin N.H., Fishman R.A. (1976). Neurologic disorders in renal failure (first of two parts). N. Engl. J. Med..

[20] Raskin N.H., Fishman R.A. (1976). Neurologic disorders in renal failure (second of two parts). N. Engl. J. Med..

[21] Marini S., Georgakis M.K., Anderson C.D. (2021). Interactions Between Kidney Function and Cerebrovascular Disease: Vessel Pathology That Fires Together Wires Together. Front. Neurol..

[22] Bobot M., Bruyat A., Thomas L., Fernandez S., Brodovitch A., Boucraut J., Burtey S., Nail V., Guillet B., Hache G. (2025). Blood-brain barrier permeability in CKD: Link with inflammation and cognitive and mood impairment in rats. Behav. Brain Res..

[23] Sen P., Sittig T., Hamers J., d’Ambrosio L., Ornek I., Zhang J., Shashikadze B., Stöckl J.B., Bachter M., Bierschenk S. (2026). From kidney injury to cardiac dysfunction: The central role of oxidative stress in diabetes and CKD. Basic Res. Cardiol..

[24] Hernandez L., Ward L.J., Arefin S., Ebert T., Laucyte-Cibulskiene A., Heimbürger O., Barany P., Wennberg L., Stenvinkel P., GOING-FWD Collaborators (2022). Blood-brain barrier and gut barrier dysfunction in chronic kidney disease with a focus on circulating biomarkers and tight junction proteins. Sci. Rep..

[25] Scheppach J.B., Wu A., Gottesman R.F., Mosley T.H., Arsiwala-Scheppach L.T., Knopman D.S., Grams M.E., Sharrett A.R., Coresh J., Koton S. (2023). Association of kidney function measures with signs of neurodegeneration and small vessel disease on brain magnetic resonance imaging: The Atherosclerosis Risk in Communities (ARIC) Study. Am. J. Kidney Dis..

[26] Martinez-Vea A., Salvadó E., Bardají A., Gutierrez C., Ramos A., García C., Compte T., Peralta C., Broch M., Pastor R. (2006). Silent cerebral white matter lesions and their relationship with vascular risk factors in middle-aged predialysis patients with CKD. Am. J. Kidney Dis..

[27] Bi R., Wei Y., Li P., Peng H., Alizadeh M., Hu B., Li Y. (2025). Associations of cerebral small vessel disease and chronic kidney disease in patients with acute ischemic stroke. J. Am. Heart Assoc..

[28] Nash P.S., Fandler-Höfler S., Ambler G., Zhang W., Ozkan H., Locatelli M., Du Y., Obergottsberger L., Wünsch G., Jäger H.R. (2024). Associations of cerebral small vessel disease and chronic kidney disease in patients with acute intracerebral hemorrhage: A cross-sectional study. Neurology.

[29] Hein Z.M., Che Mohd Nassir C.M.N. (2026). Perivascular pathology, not macrovascular complexity, governs glymphatic-related dysfunction in preclinical cerebral small vessel disease. Sci. Rep..

[30] Zhang K., Kan C., Han F., Fan Q., Wang Y., Li X., Pan R., Guo Z., Hou N., Sun X. (2026). Global trends and projections in chronic kidney disease burden from diabetes, hypertension, and glomerulonephritis: A population-based study. Kidney Blood Press. Res..

[31] Kim K.S., Park S.W., Cho Y.W., Kim S.K. (2018). Higher prevalence and progression rate of chronic kidney disease in elderly patients with type 2 diabetes mellitus. Diabetes Metab. J..

[32] Li Y., Jiang Y., Gao B., Liu N., Zhang Y., Zhou H., Song Q., Wang N., Miao Y. (2025). Regional high iron deposition on brain quantitative susceptibility mapping correlates with cognitive decline in chronic kidney disease patients. Brain Imaging Behav..

[33] Ward R.J., Zucca F.A., Duyn J.H., Crichton R.R., Zecca L. (2014). The role of iron in brain ageing and neurodegenerative disorders. Lancet Neurol..

[34] Qian J., Wang X., Cao J., Zhang W., Lu C., Chen X. (2021). Dihydromyricetin attenuates D-galactose-induced brain aging of mice via inhibiting oxidative stress and neuroinflammation. Neurosci. Lett..

[35] Neyra Chauca J.M., VanDyck M.V., Espinoza Santana A., Martínez G.G.R., Romero Vega K.A., García Quintana N., Sánchez V.F. (2026). Microvascular failure in the aging brain: Converging pathways of oxidative stress, inflammation, and endothelial decline. Biomedicines.

[36] Yu J., Li Y., Zhu B., Shen J., Miao L. (2025). Research progress on the kidney-gut-brain axis in brain dysfunction in maintenance hemodialysis patients. Front. Med..

[37] Andrews T.D., Day G.S., Irani S.R., Kanekiyo T., Hickson L.J. (2025). Uremic toxins, CKD, and cognitive dysfunction. J. Am. Soc. Nephrol..

[38] Hsieh C.-C., Lu K.-C., Huang C.-L., Wang J.-J., Yeh T.-Y., Lin S.-M., Chung Y.-L., Hou Y.-C., Huang Y.-S. (2025). Indoxyl sulfate is associated with cognitive impairment in ESRD patients by activating the extrinsic apoptosis in the neuronal cells during differentiating process. Int. J. Med. Sci..

[39] Bobot M., Guedj E., Resseguier N., Faraut J., Garrigue P., Nail V., Hache G., Gonzalez S., McKay N., Vial R. (2024). Increased blood-brain barrier permeability and cognitive impairment in patients with ESKD. Kidney Int. Rep..

[40] Sánchez-Ospina D., Mas-Fontao S., Gracia-Iguacel C., Avello A., González de Rivera M., Mujika-Marticorena M., Gonzalez-Parra E. (2024). Displacing the burden: A review of protein-bound uremic toxin clearance strategies in chronic kidney disease. J. Clin. Med..

[41] Yang J., Ke G., Liao Y., Guo Y., Gao X. (2022). Efficacy of medium cut-off dialyzers and comparison with high-flux dialyzers in patients on maintenance hemodialysis: A systematic review and meta-analysis. Ther. Apher. Dial..

[42] Zhu Y., Li J., Lu H., Shi Z., Wang X. (2024). Effect of hemodiafiltration and hemodialysis on mortality of patients with end-stage kidney disease: A meta-analysis. BMC Nephrol..

[43] Zhao Y., Gan L., Niu Q., Ni M., Zuo L. (2022). Efficacy and safety of expanded hemodialysis in hemodialysis patients: A meta-analysis and systematic review. Ren. Fail..

[44] Evans A.R., Zhao X., Ernst G.L., Ortiz-Garcia J., Dunn I.F., Burke J. (2024). Dialysis disequilibrium syndrome in neurosurgery: Literature review and illustrative case example. Geroscience.

[45] Polinder-Bos H.A., Vállez García D., Kuipers J., Elting J.W.J., Aries M.J.H., Krijnen W.P., Groen H., Willemsen A.T.M., van Laar P.J., Strijkert F. (2018). Hemodialysis induces an acute decline in cerebral blood flow in elderly patients. J. Am. Soc. Nephrol..

[46] Guo Y., Cui W., Ye P., Luo Y. (2024). Association between cerebral blood flow variation and cognitive decline in older patients undergoing hemodialysis. Front. Aging Neurosci..

[47] Kanbay M., Ertuglu L.A., Afsar B., Ozdogan E., Siriopol D., Covic A., Basile C., Ortiz A. (2020). An update review of intradialytic hypotension: Concept, risk factors, clinical implications and management. Clin. Kidney J..

[48] Etgen T., Chonchol M., Förstl H., Sander D. (2012). Chronic kidney disease and cognitive impairment: A systematic review and meta-analysis. Am. J. Nephrol..

[49] O’Lone E., Connors M., Masson P., Wu S., Kelly P.J., Gillespie D., Parker D., Whiteley W., Strippoli G.F.M., Palmer S.C. (2016). Cognition in people with end-stage kidney disease treated with hemodialysis: A systematic review and meta-analysis. Am. J. Kidney Dis..

[50] Chan F.H.F., Goh Z.Z.S., Zhu X., Tudor Car L., Newman S., Khan B.A., Griva K. (2023). Subjective cognitive complaints in end-stage renal disease: A systematic review and meta-analysis. Health Psychol. Rev..

[51] Tian X., Guo X., Xia X., Yu H., Li X., Jiang A. (2019). The comparison of cognitive function and risk of dementia in CKD patients under peritoneal dialysis and hemodialysis: A PRISMA-compliant systematic review and meta-analysis. Medicine.

[52] Oh H., Mo J., Seo W. (2019). Correlates of cognitive impairment in patients with chronic kidney failure on haemodialysis: Systematic review and meta-analysis. J. Adv. Nurs..

[53] Odagiri G., Sugawara N., Kikuchi A., Takahashi I., Umeda T., Saitoh H., Yasui-Furukori N., Kaneko S. (2011). Cognitive function among hemodialysis patients in Japan. Ann. Gen. Psychiatry.

[54] Cavanaugh K.L., Osborn C.Y., Tentori F., Rothman R.L., Ikizler T.A., Wallston K.A. (2015). Performance of a brief survey to assess health literacy in patients receiving hemodialysis. Clin. Kidney J..

[55] Chan F.H.F., Newman S., Khan B.A., Griva K. (2023). Prevalence and trajectories of subjective cognitive complaints and implications for patient outcomes: A prospective study of haemodialysis patients. Br. J. Health Psychol..

[56] Bolignano D., Simeoni M., Hafez G., Pepin M., Gallo A., Altieri M., Liabeuf S., Giannakou K., De A., Capasso G. (2024). Cognitive impairment in CKD patients: A guidance document by the CONNECT network. Clin. Kidney J..

[57] Thancharoen O., Waleekhachonloet O., Limwattananon C., Anutrakulchai S. (2020). Cognitive impairment, quality of life and healthcare utilization in patients with chronic kidney disease stages 3 to 5. Nephrology.

[58] Chan F.H.F., Sim P., Lim P.X.H., Khan B.A., Choo J.C.J., Griva K. (2024). Screening for cognitive symptoms in dialysis patients with an extended version of Kidney Disease Quality of Life Cognitive Function subscale (KDQOL-CF): A validation study. BMC Nephrol..

[59] van Zwieten A., Wong G., Ruospo M., Palmer S.C., Barulli M.R., Iurillo A., Saglimbene V., Natale P., Gargano L., Murgo M. (2018). Prevalence and patterns of cognitive impairment in adult hemodialysis patients: The COGNITIVE-HD study. Nephrol. Dial. Transplant..

[60] Drew D.A., Tighiouart H., Rollins J., Duncan S., Babroudi S., Scott T., Weiner D.E., Sarnak M.J. (2020). Evaluation of screening tests for cognitive impairment in patients receiving maintenance hemodialysis. J. Am. Soc. Nephrol..

[61] Gautam S., Kiran U.V. (2025). Co-occurrence of cognitive dysfunction and depressive disorders in hemodialysis patients: Demographic patterns and unmet diagnostic needs. Cureus.

[62] Paré M., Zola N., Coudert L., Phaneuf J.-C., Lavallée R., Caplette-Gingras A., Fortier C., Agharazii M. (2025). A mixed-methods protocol to explore psychological distress and psychosocial needs along the continuum of CKD care: A single healthcare network. BMC Nephrol..

[63] Badrkhahan S.Z., Ala M., Fakhrzadeh H., Yaghoobi A., Mirzamohamadi S., Arzaghi S.M., Shahabi S., Sharifi F., Ostovar A., Fahimfar N. (2023). The prevalence and predictors of geriatric giants in community-dwelling older adults: A cross-sectional study from the Middle East. Sci. Rep..

[64] Cassidy K.-L., Rector N.A. (2008). The silent geriatric giant: Anxiety disorders in late life. Geriatr. Aging.

[65] World Health Organization (WHO) Mental Health of Older Adults. https://www.who.int/news-room/fact-sheets/detail/mental-health-of-older-adults.

[66] Yesavage J.A., Brink T.L., Rose T.L., Lum O., Huang V., Adey M., Leirer V.O. (1982). Development and validation of a geriatric depression screening scale: A preliminary report. J. Psychiatr. Res..

[67] Pachana N.A., Byrne G.J., Siddle H., Koloski N., Harley E., Arnold E. (2007). Development and validation of the Geriatric Anxiety Inventory. Int. Psychogeriatr..

[68] Dow B., Lin X., Pachana N.A., Bryant C., LoGiudice D., Goh A.M.Y., Haralambous B. (2018). Reliability, concurrent validity, and cultural adaptation of the Geriatric Depression Scale and the Geriatric Anxiety Inventory for detecting depression and anxiety symptoms among older Chinese immigrants: An Australian study. Int. Psychogeriatr..

[69] Ganguli M., Snitz B.E., Lee C.-W., Vanderbilt J., Saxton J.A., Chang C.-C.H. (2010). Age and education effects and norms on a cognitive test battery from a population-based cohort: The Monongahela-Youghiogheny Healthy Aging Team. Aging Ment. Health.

[70] Karstens A.J., Christianson T.J., Lundt E.S., Machulda M.M., Mielke M.M., Fields J.A., Kremers W.K., Graff-Radford J., Vemuri P., Jack C.R. (2024). Mayo normative studies: Regression-based normative data for ages 30–91 years with a focus on the Boston Naming Test, Trail Making Test and Category Fluency. J. Int. Neuropsychol. Soc..

[71] Tiffin-Richards F.E., Costa A.S., Holschbach B., Frank R.D., Vassiliadou A., Krüger T., Kuckuck K., Gross T., Eitner F., Floege J. (2014). The Montreal Cognitive Assessment (MoCA)—A sensitive screening instrument for detecting cognitive impairment in chronic hemodialysis patients. PLoS ONE.

[72] Classon E., van den Hurk W., Lyth J., Johansson M.M. (2022). Montreal Cognitive Assessment: Normative data for cognitively healthy Swedish 80- to 94-year-olds. J. Alzheimer’s Dis..

[73] Scheenen M.E., van den Brink R.H.S., Konstantinidou S., Lugtenburg A., Spit J., Hendriks G.-J., Naarding P., de Vent N.R., Kessels R.P.C., Oude Voshaar R.C. (2025). Montreal Cognitive Assessment (MoCA) norms for older patients with a depressive disorder. Med. Sci..

[74] Ling T.-C., Chang C.-C., Li C.-Y., Sung J.-M., Sun C.-Y., Tsai K.-J., Cheng Y.-Y., Wu J.-L., Kuo Y.-T., Chang Y.-T. (2022). Development and validation of the dialysis dementia risk score: A retrospective, population-based, nested case-control study. Eur. J. Neurol..

[75] Lai G., Bevilacqua L., Giuliani M.E., Bigossi G., Marcozzi S., Casoli T., Abbrescia P., Frigeri A., Malavolta M., Balietti M. (2025). The aging choroid plexus and its relationship with gut dysbiosis and Klotho decline: Possible intervention strategies. Geroscience.

[76] Gregory A.C., Zablocki O., Zayed A.A., Howell A., Bolduc B., Sullivan M.B. (2020). The gut virome database reveals age-dependent patterns of virome diversity in the human gut. Cell Host Microbe.

[77] Mayneris-Perxachs J., Castells-Nobau A., Arnoriaga-Rodríguez M., Garre-Olmo J., Puig J., Ramos R., Martínez-Hernández F., Burokas A., Coll C., Moreno-Navarrete J.M. (2022). Caudovirales bacteriophages are associated with improved executive function and memory in flies, mice, and humans. Cell Host Microbe.

[78] Johansen J., Atarashi K., Arai Y., Hirose N., Sørensen S.J., Vatanen T., Knip M., Honda K., Xavier R.J., Rasmussen S. (2023). Centenarians have a diverse gut virome with the potential to modulate metabolism and promote healthy lifespan. Nat. Microbiol..

[79] Shuai M., Fu Y., Zhong H.-L., Gou W., Jiang Z., Liang Y., Miao Z., Xu J.-J., Huynh T., Wahlqvist M.L. (2022). Mapping the human gut mycobiome in middle-aged and elderly adults: Multiomics insights and implications for host metabolic health. Gut.

[80] Lai S., Yan Y., Pu Y., Lin S., Qiu J.-G., Jiang B.-H., Keller M.I., Wang M., Bork P., Chen W.-H. (2023). Enterotypes of the human gut mycobiome. Microbiome.

[81] Mihajlovski A., Doré J., Levenez F., Alric M., Brugère J.-F. (2010). Molecular evaluation of the human gut methanogenic archaeal microbiota reveals an age-associated increase of the diversity. Environ. Microbiol. Rep..

[82] Dridi B., Henry M., Richet H., Raoult D., Drancourt M. (2012). Age-related prevalence of *Methanomassiliicoccus luminyensis* in the human gut microbiome. APMIS.

[83] Wu L., Xie X., Li Y., Liang T., Zhong H., Yang L., Xi Y., Zhang J., Ding Y., Wu Q. (2022). Gut microbiota as an antioxidant system in centenarians associated with high antioxidant activities of gut-resident *Lactobacillus*. npj Biofilms Microbiomes.

[84] Pu L., Pang S., Mu W., Chen X., Zou Y., Wang Y., Ding Y., Yan Q., Huang Y., Chen X. (2024). The gut mycobiome signatures in long-lived populations. iScience.

[85] Mohammadzadeh R., Mahnert A., Shinde T., Kumpitsch C., Weinberger V., Schmidt H., Moissl-Eichinger C. (2025). Age-related dynamics of predominant methanogenic archaea in the human gut microbiome. BMC Microbiol..

[86] Chen H., Wang J., Ouyang Q., Peng X., Yu Z., Wang J., Huang J. (2023). Alterations of gut microbes and their correlation with clinical features in middle and end-stages chronic kidney disease. Front. Cell. Infect. Microbiol..

[87] Zhang P., Wang X., Li S., Cao X., Zou J., Fang Y., Shi Y., Xiang F., Shen B., Li Y. (2023). Metagenome-wide analysis uncovers gut microbial signatures and implicates taxon-specific functions in end-stage renal disease. Genome Biol..

[88] Zhao J., Ning X., Liu B., Dong R., Bai M., Sun S. (2021). Specific alterations in gut microbiota in patients with chronic kidney disease: An updated systematic review. Ren. Fail..

[89] Stanford J., Charlton K., Stefoska-Needham A., Ibrahim R., Lambert K. (2020). The gut microbiota profile of adults with kidney disease and kidney stones: A systematic review of the literature. BMC Nephrol..

[90] Candeliere F., Simone M., Leonardi A., Rossi M., Amaretti A., Raimondi S. (2022). Indole and p-cresol in feces of healthy subjects: Concentration, kinetics, and correlation with microbiome. Front. Mol. Med..

[91] Amini Khiabani S., Asgharzadeh M., Samadi Kafil H. (2023). Chronic kidney disease and gut microbiota. Heliyon.

[92] Laiola M., Koppe L., Larabi A., Thirion F., Lange C., Quinquis B., David A., Le Chatelier E., Benoit B., Sequino G. (2025). Toxic microbiome and progression of chronic kidney disease: Insights from a longitudinal CKD-Microbiome Study. Gut.

[93] Wyczalkowska-Tomasik A., Czarkowska-Paczek B., Giebultowicz J., Wroczynski P., Paczek L. (2017). Age-dependent increase in serum levels of indoxyl sulphate and p-cresol sulphate is not related to their precursors: Tryptophan and tyrosine. Geriatr. Gerontol. Int..

[94] Wong J., Piceno Y.M., DeSantis T.Z., Pahl M., Andersen G.L., Vaziri N.D. (2014). Expansion of urease- and uricase-containing, indole- and p-cresol-forming and contraction of short-chain fatty acid-producing intestinal microbiota in ESRD. Am. J. Nephrol..

[95] Karbowska M., Hermanowicz J.M., Tankiewicz-Kwedlo A., Kalaska B., Kaminski T.W., Nosek K., Wisniewska R.J., Pawlak D. (2020). Neurobehavioral effects of uremic toxin-indoxyl sulfate in the rat model. Sci. Rep..

[96] Iwata K., Watanabe H., Morisaki T., Matsuzaki T., Ohmura T., Hamada A., Saito H. (2007). Involvement of indoxyl sulfate in renal and central nervous system toxicities during cisplatin-induced acute renal failure. Pharm. Res..

[97] Łukawski K., Raszewski G., Czuczwar S.J. (2024). Effects of the uremic toxin indoxyl sulfate on seizure activity, learning and brain oxidative stress parameters in mice. Neurosci. Lett..

[98] Adesso S., Magnus T., Cuzzocrea S., Campolo M., Rissiek B., Paciello O., Autore G., Pinto A., Marzocco S. (2017). Indoxyl sulfate affects glial function increasing oxidative stress and neuroinflammation in chronic kidney disease: Interaction between astrocytes and microglia. Front. Pharmacol..

[99] Vaziri N.D., Wong J., Pahl M., Piceno Y.M., Yuan J., DeSantis T.Z., Ni Z., Nguyen T.-H., Andersen G.L. (2013). Chronic kidney disease alters intestinal microbial flora. Kidney Int..

[100] Sampaio-Maia B., Simões-Silva L., Pestana M., Araujo R., Soares-Silva I.J. (2016). The role of the gut microbiome on chronic kidney disease. Adv. Appl. Microbiol..

[101] Cao Q., Shen M., Li R., Liu Y., Zeng Z., Zhou J., Niu D., Zhang Q., Wang R., Yao J. (2025). Elucidating the specific mechanisms of the gut-brain axis: The short-chain fatty acids-microglia pathway. J. Neuroinflamm..

[102] Fock E., Parnova R. (2023). Mechanisms of blood-brain barrier protection by microbiota-derived short-chain fatty acids. Cells.

[103] Moțățăianu A., Șerban G., Andone S. (2023). The role of short-chain fatty acids in microbiota-gut-brain cross-talk with a focus on amyotrophic lateral sclerosis: A systematic review. Int. J. Mol. Sci..

[104] Lu C., Wang X., Chen X., Qin T., Ye P., Liu J., Wang S., Luo W. (2025). Causal analysis between gut microbes, aging indicator, and age-related disease, involving the discovery and validation of biomarkers. Aging Cell.

[105] Zhang S., Easwaran M., Elafify M., Mahmoud A.A., Wang X., Ahn J. (2025). Prophages and their interactions with lytic phages in the human gut microbiota and their impact on microbial diversity, gut health, and disease. Appl. Environ. Microbiol..

[106] Chaudhari D.S., Jain S., Yata V.K., Mishra S.P., Kumar A., Fraser A., Kociolek J., Dangiolo M., Smith A., Golden A. (2023). Unique trans-kingdom microbiome structural and functional signatures predict cognitive decline in older adults. Geroscience.

[107] Ghorbani M., Ferreira D., Maioli S. (2023). A metagenomic study of gut viral markers in amyloid-positive Alzheimer’s disease patients. Alzheimer’s Res. Ther..

[108] Chen W., Guo R., Zhang W., Yan Q., Wang X., Chen R., Hu X., Liang J., Xing G., Xu D. (2026). Alterations of the gut virome in patients with Parkinson’s disease. J. Gerontol. A Biol. Sci. Med. Sci..

[109] Zhang P., Guo R., Ma S., Jiang H., Yan Q., Li S., Wang K., Deng J., Zhang Y., Zhang Y. (2025). A metagenome-wide study of the gut virome in chronic kidney disease. Theranostics.

[110] Badillo-Pazmay G.V., Fortunato C., Cianfruglia L., Novazzi F., Spezia P.G., Rosa L., Limongi D., Prezioso C., D’Argenio V., Scudiero O. (2025). The gut and circulating virome: Emerging players in aging and longevity. Front. Aging.

[111] Hallen-Adams H.E., Suhr M.J. (2017). Fungi in the healthy human gastrointestinal tract. Virulence.

[112] Hu J., Wei S., Gu Y., Wang Y., Feng Y., Sheng J., Hu L., Gu C., Jiang P., Tian Y. (2022). Gut mycobiome in patients with chronic kidney disease was altered and associated with immunological profiles. Front. Immunol..

[113] Qiu J., Zhao L., Cheng Y., Chen Q., Xu Y., Lu Y., Gao J., Lei W., Yan C., Ling Z. (2023). Exploring the gut mycobiome: Differential composition and clinical associations in hypertension, chronic kidney disease, and their comorbidity. Front. Immunol..

[114] Ren Y., Chen L., Guo R., Ma S., Li S., Zhang Y., Jiang H., Shi H., Zhang P. (2024). Altered gut mycobiome in patients with end-stage renal disease and its correlations with serum and fecal metabolomes. J. Transl. Med..

[115] Nie T., Li J., You L., Wu Q. (2025). Environmental mycotoxins: A potential etiological factor for neurodegenerative diseases?. Toxicology.

[116] Nagpal R., Neth B.J., Wang S., Mishra S.P., Craft S., Yadav H. (2020). Gut mycobiome and its interaction with diet, gut bacteria and Alzheimer’s disease markers in subjects with mild cognitive impairment: A pilot study. EBioMedicine.

[117] Umamahesan C., Pilcicka A., Yick J., Baker K., Smith M., Taylor D., Ma Y., Mullish B.H., Marchesi J.R., Gilbert S. (2025). Interplay of constipation, intestinal barrier dysfunction and fungal exposome in aetiopathogenesis of Parkinson’s disease: Hypothesis with supportive data. Biochem. J..

[118] Mafra D., Alvarenga L., Cardozo L.F.M.F., Schultz J., Rosado A.S., Borges N.A. (2025). Gut microbiota and NLRP3 inflammasome activation in hemodialysis patients: Exploring the link with systemic inflammation. Mol. Biol. Rep..

[119] Kemp J.A., Schultz J., Modolon F., Ribeiro-Alves M., Rosado A.S., Mafra D. (2025). Is there a correlation between TMAO plasma levels and archaea in the gut of patients undergoing hemodialysis?. Int. Urol. Nephrol..

[120] Fumagalli A., Castells-Nobau A., Trivedi D., Garre-Olmo J., Puig J., Ramos R., Ramió-Torrentà L., Pérez-Brocal V., Moya A., Swann J. (2025). Archaea methanogens are associated with cognitive performance through the shaping of gut microbiota, butyrate and histidine metabolism. Gut Microbes.

[121] Duru I.C., Lecomte A., Shishido T.K., Laine P., Suppula J., Paulin L., Scheperjans F., Pereira P.A.B., Auvinen P. (2024). Metagenome-assembled microbial genomes from Parkinson’s disease fecal samples. Sci. Rep..

[122] Shoubridge A.P., Carpenter L., Flynn E., Papanicolas L.E., Collins J., Gordon D., Lynn D.J., Whitehead C., Leong L.E.X., Cations M. (2025). Severe cognitive decline in long-term care is related to gut microbiome production of metabolites involved in neurotransmission, immunomodulation, and autophagy. J. Gerontol. A Biol. Sci. Med. Sci..

[123] Tanaka K., Saito H., Iwasaki T., Oda A., Watanabe S., Kanno M., Kimura H., Shimabukuro M., Asahi K., Watanabe T. (2021). Association between serum potassium levels and adverse outcomes in chronic kidney disease: The Fukushima CKD cohort study. Clin. Exp. Nephrol..

[124] Suzuki Y., Misaka T., Sato Y., Okochi S., Ogawara R., Ichimura S., Yokokawa T., Sato A., Shimizu T., Sato T. (2025). Association between serum potassium variability during hospitalization and clinical outcomes in patients with heart failure. Eur. J. Intern. Med..

[125] Iqbal I.M., Obaid M., Haider A.S., Asif A., Ul Haq Z., Salman M., Rehman I. (2025). Prevalence of electrolyte imbalances in critically ill medical intensive care unit patients and their association with clinical outcomes. Cureus.

[126] Guo Y., Qiu Y., Xue T., Yan P., Zhao W., Wang M., Liu C., Zhang N. (2024). Association between admission baseline blood potassium levels and all-cause mortality in patients with acute kidney injury combined with sepsis: A retrospective cohort study. PLoS ONE.

[127] Arnold R., Pussell B.A., Howells J., Grinius V., Kiernan M.C., Lin C.S.-Y., Krishnan A.V. (2014). Evidence for a causal relationship between hyperkalaemia and axonal dysfunction in end-stage kidney disease. Clin. Neurophysiol..

[128] Kiernan M.C., Walters R.J.L., Andersen K.V., Taube D., Murray N.M.F., Bostock H. (2002). Nerve excitability changes in chronic renal failure indicate membrane depolarization due to hyperkalaemia. Brain.

[129] Babaker M.A., Alazabi N.I., Yousef E.M., Haredy S.A., Algohary A.M., Mansour D.F., Ahmed-Farid O.A. (2026). Taurine mitigates spironolactone-induced hyperkalemia and cognitive dysfunction: A biochemical and histological study in a rat model. Appl. Biochem. Biotechnol..

[130] Melekoglu E., Samur F.G. (2023). Dietary strategies for gut-derived protein-bound uremic toxins and cardio-metabolic risk factors in chronic kidney disease: A focus on dietary fibers. Crit. Rev. Food Sci. Nutr..

[131] St-Jules D.E., Goldfarb D.S., Sevick M.A. (2016). Nutrient non-equivalence: Does restricting high-potassium plant foods help to prevent hyperkalemia in hemodialysis patients?. J. Ren. Nutr..

[132] Joshi S., Shah S., Kalantar-Zadeh K. (2019). Adequacy of plant-based proteins in chronic kidney disease. J. Ren. Nutr..

[133] Avesani C.M., Heimbürger O., Rubin C., Sallstrom T., Fáxen-Irving G., Lindholm B., Stenvinkel P. (2024). Plant-based diet in hyperkalemic chronic kidney disease patients receiving sodium zirconium cyclosilicate: A feasibility clinical trial. Am. J. Clin. Nutr..

[134] Michail A., Andreou E. (2025). A plant-dominant low-protein diet in chronic kidney disease management: A narrative review with considerations for Cyprus. Nutrients.

[135] Zarantonello D., Brunori G. (2023). The Role of Plant-Based Diets in Preventing and Mitigating Chronic Kidney Disease: More Light than Shadows. J. Clin. Med..

[136] Shannon O.M., Ranson J.M., Gregory S., Macpherson H., Milte C., Lentjes M., Mulligan A., McEvoy C., Griffiths A., Matu J. (2023). Mediterranean diet adherence is associated with lower dementia risk, independent of genetic predisposition: Findings from the UK Biobank prospective cohort study. BMC Med..

[137] Chen H., Dhana K., Huang Y., Huang L., Tao Y., Liu X., Melo van Lent D., Zheng Y., Ascherio A., Willett W. (2023). Association of the Mediterranean dietary approaches to stop hypertension intervention for neurodegenerative delay (MIND) diet with the risk of dementia. JAMA Psychiatry.

[138] Babitt J.L., Berns J.S., Bozkurt B., Cheung Khedairy R.S., Cuevas Y., Effa E.E., Eisenga M.F., Fishbane S., Ginzburg Y.Z., Haase V.H. (2026). Executive summary of the KDIGO 2026 clinical practice guideline for the management of anemia in chronic kidney disease (CKD). Kidney Int..

[139] Valletta M., Vetrano D.L., Qiu C., Canevelli M., Miccoli E., Andersson S., Fredolini C., Bruno G., Winblad B., Fratiglioni L. (2026). Anemia and blood biomarkers of Alzheimer disease in dementia development. JAMA Netw. Open.

[140] Choi S., O’Neil S.H., Joshi A.A., Li J., Bush A.M., Coates T.D., Leahy R.M., Wood J.C. (2019). Anemia predicts lower white matter volume and cognitive performance in sickle and non-sickle cell anemia syndrome. Am. J. Hematol..

[141] Hanna R.M., Streja E., Kalantar-Zadeh K. (2021). Burden of anemia in chronic kidney disease: Beyond erythropoietin. Adv. Ther..

[142] Greenwood S.A., Beckley-Hoelscher N., Asgari E., Ayis S., Baker L.A., Banerjee D., Bhandari S., Bramham K., Chilcot J., Burton J. (2022). The effect of intravenous iron supplementation on exercise capacity in iron-deficient but not anaemic patients with chronic kidney disease: Study design and baseline data for a multicentre prospective double-blind randomised controlled trial. BMC Nephrol..

[143] Liu H., Wu W., Luo Y. (2023). Oral and intravenous iron treatment alter the gut microbiome differentially in dialysis patients. Int. Urol. Nephrol..

[144] Shearer J., Shah S., MacInnis M.J., Shen-Tu G., Mu C. (2024). Dose-responsive effects of iron supplementation on the gut microbiota in middle-aged women. Nutrients.

[145] Rozen-Zvi B., Gafter-Gvili A., Paul M., Leibovici L., Shpilberg O., Gafter U. (2008). Intravenous versus oral iron supplementation for the treatment of anemia in CKD: Systematic review and meta-analysis. Am. J. Kidney Dis..

[146] Li Y., Han M., Song J., Liu S., Wang Y., Su X., Wei K., Xu Z., Li H., Wang Z. (2022). The prebiotic effects of soluble dietary fiber mixture on renal anemia and the gut microbiota in end-stage renal disease patients on maintenance hemodialysis: A prospective, randomized, placebo-controlled study. J. Transl. Med..

[147] Patel P., Hashmi M.F. (2026). Chronic kidney disease-mineral bone disorder (CKD-MBD). StatPearls [Internet].

[148] Li T., Xie Y., Bowe B., Xian H., Al-Aly Z. (2017). Serum phosphorus levels and risk of incident dementia. PLoS ONE.

[149] Rodríguez-Ortiz M.E., Jurado-Montoya D., Valdés-Díaz K., García-Sáez R.M., Torralbo A.I., Obrero T., Vidal-Jiménez V., Jiménez M.J., Carmona A., Guerrero F. (2024). Cognitive impairment related to chronic kidney disease is associated with a decreased abundance of membrane-bound Klotho in the cerebral cortex. Int. J. Mol. Sci..

[150] Lampen A., Lachenmeier D.W., Diel P., Ensenauer R., Frommherz L., Guth S., Humpf H.-U., Kulling S.E., Villar-Fernández M.A., Wätjen W. (2026). Dietary phosphate exposure-strategies to protect vulnerable population groups. Arch. Toxicol..

[151] Biruete A., Chen N.X., Metzger C.E., Srinivasan S., O’Neill K., Fallen P.B., Fonseca A., Wilson H.E., de Loor H., Evenepoel P. (2023). The dietary fiber inulin slows progression of chronic kidney disease-mineral bone disorder (CKD-MBD) in a rat model of CKD. JBMR Plus.

[152] Lai S., Molfino A., Testorio M., Perrotta A.M., Currado A., Pintus G., Pietrucci D., Unida V., La Rocca D., Biocca S. (2019). Effect of low-protein diet and inulin on microbiota and clinical parameters in patients with chronic kidney disease. Nutrients.

[153] Zhang Y., Hu X.-Y., Yang S.-Y., Hu Y.-C., Duan K. (2024). Effects of resistant starch supplementation on renal function and inflammatory markers in patients with chronic kidney disease: A meta-analysis of randomized controlled trials. Ren. Fail..

[154] Du X., Wu J., Gao C., Tan Q., Xu Y. (2022). Effects of resistant starch on patients with chronic kidney disease: A systematic review and meta-analysis. J. Diabetes Res..

[155] Hu B., Wang Y., Yu L., Cao L., Liu S., Zhong L., Wang G., Qiu X., Hou H. (2025). Biomimetic wrinkled prebiotic microspheres with enhanced intestinal retention for hyperphosphatemia and vascular calcification. Sci. Adv..

[156] Fouque D., Kalantar-Zadeh K., Kopple J., Cano N., Chauveau P., Cuppari L., Franch H., Guarnieri G., Ikizler T.A., Kaysen G. (2008). A proposed nomenclature and diagnostic criteria for protein-energy wasting in acute and chronic kidney disease. Kidney Int..

[157] Carrero J.J., Thomas F., Nagy K., Arogundade F., Avesani C.M., Chan M., Chmielewski M., Cordeiro A.C., Espinosa-Cuevas A., Fiaccadori E. (2018). Global prevalence of protein-energy wasting in kidney disease: A meta-analysis of contemporary observational studies from the International Society of Renal Nutrition and Metabolism. J. Ren. Nutr..

[158] Hu J., Zhong X., Liu Y., Yan J., Zhou D., Qin D., Xiao X., Zheng Y., Wen L., Tan R. (2022). Correlation between intestinal flora disruption and protein-energy wasting in patients with end-stage renal disease. BMC Nephrol..

[159] Bi X., Liu Y., Yao L., Ling L., Lu J., Hu C., Ding W. (2024). Gut microbiota dysbiosis and protein energy wasting in patients on hemodialysis: An observational longitudinal study. Front. Nutr..

[160] De Souza da Costa Brum I., de Luca Corrêa H., Rosa T.S., de Souza J.F., Fouque D., Mafra D. (2025). Low-protein diet adherence and CKD progression during long-term follow-up. Nephrol. Dial. Transplant..

[161] Kim S.M., Jung J.Y. (2020). Nutritional management in patients with chronic kidney disease. Korean J. Intern. Med..

[162] Hahn D., Hodson E.M., Fouque D. (2020). Low protein diets for non-diabetic adults with chronic kidney disease. Cochrane Database Syst. Rev..

[163] Ikizler T.A. (2013). Optimal nutrition in hemodialysis patients. Adv. Chronic Kidney Dis..

[164] Ebersolt M., Machado T.S., Mallmann C., Mc-Kay N., Dou L., Bouchouareb D., Brunet P., Burtey S., Sallée M. (2022). Protein/fiber index modulates uremic toxin concentrations in hemodialysis patients. Toxins.

[165] Ozcan B., Ikizler T.A. (2026). Portein Intake in Hemodialysis Patients Should Be Higher Than 1.2 g/Kg per Day: CON. Kidney360.

[166] De Filippis F., Pellegrini N., Vannini L., Jeffery I.B., La Storia A., Laghi L., Serrazanetti D.I., Di Cagno R., Ferrocino I., Lazzi C. (2016). High-level adherence to a Mediterranean diet beneficially impacts the gut microbiota and associated metabolome. Gut.

[167] David L.A., Maurice C.F., Carmody R.N., Gootenberg D.B., Button J.E., Wolfe B.E., Ling A.V., Devlin A.S., Varma Y., Fischbach M.A. (2014). Diet rapidly and reproducibly alters the human gut microbiome. Nature.

[168] Hung K.-Y., Chiou T.T.-Y., Wu C.-H., Liao Y.-C., Chen C.-N., Yang P.-H., Wang H.-J., Lee C.-T. (2017). Effects of diet intervention on body composition in the elderly with chronic kidney disease. Int. J. Med. Sci..

[169] Yang W.-C., Hsieh H.-M., Chen J.-P., Liu L.-C., Chen C.-H. (2023). Effects of a low-protein nutritional formula with dietary counseling in older adults with chronic kidney disease stages 3–5: A randomized controlled trial. BMC Nephrol..

[170] Liu J., Wan J., Chen K., He Y., Zhang W., Luo J., Li D. (2025). The relationship between protein-energy wasting and cognitive impairment in patients receiving maintenance hemodialysis. Front. Neurol..

[171] Yang X., Quan Y., Wu E., Jiang Y., Song Q., Li Y., Li Q., Sun Z., Yuan J., Zha Y. (2023). The association of cognition with protein energy wasting and synaptic transmission in chronic kidney disease. Semin. Dial..

[172] Connell E., Sami S., Khondoker M., Minihane A.M., Pontifex M.G., Müller M., McArthur S., Le Gall G., Vauzour D. (2026). Circulatory dietary and gut-derived metabolites predict early cognitive decline. Gut Microbes.

[173] Pieniazek A., Bernasinska-Slomczewska J., Gwozdzinski L. (2021). Uremic toxins and their relation with oxidative stress induced in patients with CKD. Int. J. Mol. Sci..

[174] Ticinesi A., Guerra A., Nouvenne A., Meschi T., Maggi S. (2023). Disentangling the complexity of nutrition, frailty and gut microbial pathways during aging: A focus on hippuric acid. Nutrients.

[175] Xia J., Zhang Y., Zhang S., Lu C., Huan H., Guan X. (2024). Oat dietary fiber delays the progression of chronic kidney disease in mice by modulating the gut microbiota and reducing uremic toxin levels. J. Agric. Food Chem..

[176] Rocchetti M.T., Di Iorio B.R., Vacca M., Cosola C., Marzocco S., di Bari I., Calabrese F.M., Ciarcia R., De Angelis M., Gesualdo L. (2021). Ketoanalogs’ effects on intestinal microbiota modulation and uremic toxins serum levels in chronic kidney disease (Medika2 study). J. Clin. Med..

[177] Liu X., Zhang M., Wang X., Liu P., Wang L., Li Y., Wang X., Ren F. (2022). Fecal microbiota transplantation restores normal fecal composition and delays malignant development of mild chronic kidney disease in rats. Front. Microbiol..

[178] Barba C., Soulage C.O., Caggiano G., Glorieux G., Fouque D., Koppe L. (2020). Effects of fecal microbiota transplantation on composition in mice with CKD. Toxins.

[179] Arteaga-Muller G.Y., Flores-Treviño S., Bocanegra-Ibarias P., Robles-Espino D., Garza-González E., Fabela-Valdez G.C., Camacho-Ortiz A. (2024). Changes in the progression of chronic kidney disease in patients undergoing fecal microbiota transplantation. Nutrients.

[180] Ye J., Jiang Y., Yang K., Hu J., Lu J., Tian B. (2026). Changyanning ameliorates dextran sodium sulfate-induced colitis by modulating gut microbiota inflammation and intestinal barrier in mice. Mol. Nutr. Food Res..

[181] Huang P., Hu J., Yang K., Tian B. (2025). Digestion characteristics of Changyanning formula and its regulatory effect on fecal microbiota and metabolites during in vitro fermentation. Fitoterapia.

[182] Pei T., Zhu D., Yang S., Hu R., Wang F., Zhang J., Yan S., Ju L., He Z., Han Z. (2022). *Bacteroides plebeius* improves muscle wasting in chronic kidney disease by modulating the gut-renal muscle axis. J. Cell. Mol. Med..

[183] Ni J., Yin Y., Liang P., Zheng Y., Li Y., Pang L., Zhong X., Hu J. (2025). *Faecalibacterium prausnitzii* suppresses mitophagy to alleviate muscle atrophy in chronic renal failure with protein-energy wasting. APMIS.

[184] Anegkamol W., Bowonsomsarit W., Taweevisit M., Tumwasorn S., Thongsricome T., Kaewwongse M., Pitchyangkura R., Tosukhowong P., Chuaypen N., Dissayabutra T. (2025). Synbiotics as a novel therapeutic approach for hyperphosphatemia and hyperparathyroidism in chronic kidney disease rats. Sci. Rep..

[185] Iwashita Y., Ohya M., Yashiro M., Sonou T., Kawakami K., Nakashima Y., Yano T., Iwashita Y., Mima T., Negi S. (2018). Dietary changes involving *Bifidobacterium longum* and other nutrients delays chronic kidney disease progression. Am. J. Nephrol..

[186] Haghighat N., Mohammadshahi M., Shayanpour S., Haghighizadeh M.H., Rahmdel S., Rajaei M. (2021). The effect of synbiotic and probiotic supplementation on mental health parameters in patients undergoing hemodialysis: A double-blind, randomized, placebo-controlled trial. Indian J. Nephrol..

[187] Kooshki A., Akbarzadeh R., Amin B., Tofighiyan T., Foroumandi E. (2023). Synbiotic supplement for treatment of iron deficiency anaemia in haemodialysis patients: A randomized controlled trial. Nephrology.

[188] Viramontes-Hörner D., Márquez-Sandoval F., Martín-del-Campo F., Vizmanos-Lamotte B., Sandoval-Rodríguez A., Armendáriz-Borunda J., García-Bejarano H., Renoirte-López K., García-García G. (2015). Effect of a symbiotic gel (*Lactobacillus acidophilus* + *Bifidobacterium lactis* + inulin) on presence and severity of gastrointestinal symptoms in hemodialysis patients. J. Ren. Nutr..

[189] Liu C., Yang L., Wei W., Fu P. (2024). Efficacy of probiotics/synbiotics supplementation in patients with chronic kidney disease: A systematic review and meta-analysis of randomized controlled trials. Front. Nutr..

[190] Mirzaeian S., Saraf-Bank S., Entezari M.H., Hekmatdoost A., Feizi A., Atapour A. (2020). Effects of synbiotic supplementation on microbiota-derived protein-bound uremic toxins, systemic inflammation, and biochemical parameters in patients on hemodialysis: A double-blind, placebo-controlled, randomized clinical trial. Nutrition.

[191] Mitrović M., Stanković-Popović V., Tolinački M., Golić N., Soković Bajić S., Veljović K., Nastasijević B., Soldatović I., Svorcan P., Dimković N. (2023). The impact of synbiotic treatment on the levels of gut-derived uremic toxins, inflammation, and gut microbiome of chronic kidney disease patients—A randomized trial. J. Ren. Nutr..

[192] Cedillo-Flores R., Cuevas-Budhart M.A., Cavero-Redondo I., Kappes M., Ávila-Díaz M., Paniagua R. (2025). Impact of gut microbiome modulation on uremic toxin reduction in chronic kidney disease: A systematic review and network meta-analysis. Nutrients.

[193] Favero C., Giordano L., Mihaila S.M., Masereeuw R., Ortiz A., Sanchez-Niño M.D. (2022). Postbiotics and kidney disease. Toxins.

[194] Ikizler T.A., Burrowes J.D., Byham-Gray L.D., Campbell K.L., Carrero J.J., Chan W., Fouque D., Friedman A.N., Ghaddar S., Goldstein-Fuchs D.J. (2020). KDOQI clinical practice guideline for nutrition in CKD: 2020 update. Am. J. Kidney Dis..

[195] Ertuglu L., Ikizler T.A. (2024). Nutrition management in geriatric patients with CKD. Kidney360.

[196] Porcari S., Mullish B.H., Asnicar F., Ng S.C., Zhao L., Hansen R., O’Toole P.W., Raes J., Hold G., Putignani L. (2025). International consensus statement on microbiome testing in clinical practice. Lancet Gastroenterol. Hepatol..

[197] McFarlane C., Ramos C.I., Johnson D.W., Campbell K.L. (2019). Prebiotic, probiotic, and synbiotic supplementation in chronic kidney disease: A systematic review and meta-analysis. J. Ren. Nutr..

